# In vivo effects of balanced, low molecular 6% and 10% hydroxyethyl starch compared with crystalloid volume replacement on the coagulation system in major pancreatic surgery—a sub-analysis of a prospective double-blinded, randomized controlled trial

**DOI:** 10.1371/journal.pone.0303165

**Published:** 2024-07-11

**Authors:** Alexander Eckers, Oliver Hunsicker, Claudia Spies, Felix Balzer, Kerstin Rubarth, Christian von Heymann

**Affiliations:** 1 Department of Anesthesiology and Operative Intensive Care Medicine (CCM, CVK), Charité—Universitätsmedizin Berlin, Corporate Member of Freie Universität Berlin, Humboldt-Universität zu Berlin and Berlin Institute of Health, Campus Virchow-Klinikum, Berlin, Germany; 2 Institute of Medical Informatics, Charité—Universitätsmedizin Berlin, Berlin, Germany; 3 Institute of Biometry and Clinical Epidemiology, Charité—Universitätsmedizin Berlin, Berlin, Germany; 4 Department of Anesthesia, Intensive Care Medicine, Emergency Medicine and Pain Therapy, Vivantes Klinikum im Friedrichshain, Berlin, Germany; Sant Anna Hospital: Clinica Sant’Anna, SWITZERLAND

## Abstract

**Background:**

The outcome of patients undergoing major surgery treated with HES for hemodynamic optimization is unclear. This post-hoc analysis of a randomized clinical pilot trial investigated the impact of low-molecular balanced HES solutions on the coagulation system, blood loss and transfusion requirements.

**Methods:**

The Trial was registered: EudraCT 2008-004175-22 and ethical approval was provided by the ethics committee of Berlin. Patients were randomized into three groups receiving either a 10% HES 130/0.42 solution, a 6% HES 130/0.42 solution or a crystalloid following a goal-directed hemodynamic algorithm. Endpoints were parameters of standard and viscoelastic coagulation laboratory, blood loss and transfusion requirements at baseline, at the end of surgery (EOS) and the first postoperative day (POD 1).

**Results:**

Fifty-two patients were included in the analysis (HES 10% (n = 15), HES 6% (n = 17) and crystalloid (n = 20)). Fibrinogen decreased in all groups at EOS (HES 10% 338 [298;378] to 192 [163;234] mg dl^-1^, p<0.01, HES 6% 385 [302;442] to 174 [163;224] mg dl^-1^, p<0.01, crystalloids 408 [325;458] to 313 [248;370] mg dl^-1^, p = 0.01). MCF FIBTEM was decreased for both HES groups at EOS (HES 10%: 20.5 [16.0;24.8] to 6.5 [5.0;10.8] mm, p = <0.01; HES 6% 27.0 [18.8;35.2] to 7.0 [5.0;19.0] mm, p = <0.01). These changes did not persist on POD 1 for HES 10% (rise to 16.0 [13.0;24.0] mm, p = 0.88). Blood loss was not different in the groups nor transfusion requirements.

**Conclusion:**

Our data suggest a stronger but transient effect of balanced, low-molecular HES on the coagulation system. Despite the decline of the use of artificial colloids in clinical practice, these results may help to inform clinicians who use HES solutions.

## Introduction

During major surgery, adequate fluid and volume therapy is a cornerstone to maintain hemostasis, blood volume and reduce perioperative complications [1–4]. Hydroxyethyl starch (HES) was used for years to maintain intravascular volume because of a longer volume expansion effect as compared to crystalloids [5]. Compared to older HES preparations, modern low molecular weight solutions show less side effects on the coagulation system, such as dilutional coagulopathy [6], induced von Willebrand disorder [7] or dysfunction in fibrin polymerisation [8, 9]. Most of this evidence generates from in vitro studies, which may not fully reflect the effect of the biological environment on hemostasis in the clinical setting [10, 11].

Of note the U.S. Food and Drug Administration and the European Medicines Agency (EMA) recommend to avoid HES solutions in critical ill patients since 2013 due to an increased risk for acute kidney injury and mortality [12, 13]. After strengthening this statement in 2018, the EMA now has completely lifted the approval after an ongoing clinical overuse [14, 15]. However, recent data suggest that these results must not be generalized to patients undergoing major surgery [16–18].

This subanalysis of a previously published, prospective, randomized, double-blinded study on HES and renal function in patients undergoing major abdominal surgery [19] focuses on the effect of HES solutions on the coagulation system in pancreatic surgery, for which reliable data from randomized controlled clinical trials are scarce.

We hypothesized that balanced low molecular HES solutions, irrespective of their concentration (iso oncotic 6% versus hyper oncotic 10%), and used within a goal directed hemodynamic optimization algorithm [4, 19] to improve surgical outcomes, will impair the coagulation system more severely as compared to crystalloids in the clinical setting of pancreatic surgery. Furthermore, it is hypothesized that this effect will be greater in a 10% HES than in a 6% HES solution.

## Methods

### Ethics and study registration

Ethical approval for this study (No. ZS EK 11026/09) was provided by the ethics committee of Berlin (Ethik-Kommission des Landes Berlin), Germany (Chairman Dr. C. von Dewitz) on 25^th^ February 2009. The Trial was registered: EudraCT 2008-004175-22; ClinicalTrials.gov: NCT01117649, principal investigator: Christian von Heymann, 25.02.2009.

### Setting and intervention

The presented study is a predefined subanalysis of a randomized, double blind controlled pilot trial comparing a hyperoncotic balanced 10% HES 130/0.42 solution (Tetraspan 10%^®^, B. Braun, Melsungen, Germany), an isooncotic balanced 6% HES 130/0.42 solution (Tetraspan 6%^®^, B. Braun) or a balanced crystalloid (Sterofundin ISO^®^, B. Braun) within a goal-directed hemodynamic algorithm to improve surgical outcomes in patients undergoing pancreatic surgery. This subanalysis investigates the impact of low molecular HES solutions on the coagulation system that was not reported in the previous publication of the main study. Study design, interventions and patient enrollment have been described earlier [19].

Between June 2010 and July 2012 the study was conducted in three German hospitals. Patients between 18 and 80 years, scheduled for elective surgery of the pancreatic head were enrolled. Compromised hemostasis as seen as a reduced prothrombin time ratio<60%, impaired liver function of Child Pugh C severity or a known bleeding disorder were part of the exclusion criteria. Written informed consent was obtained from all patients before surgery who were independently randomized into three groups in a 1:1:1 ratio to either receive a hyperoncotic balanced 10% HES 130/0.42 solution, an isooncotic balanced 6% HES 130/0.42 solution or a balanced crystalloid.

The perioperative care followed the standard operating procedures of each study site. For the hemodynamic management an esophageal Doppler monitored (EDM, CardioQ-ODM^TM^, Deltex Medical, Chichester, UK), goal-directed hemodynamic algorithm guided the application of the study solutions as described earlier [4, 19]. Transfusion of packed red blood cells were triggered by a hematocrit < 25% or in case of severe bleeding according to physician’s judgement.

Reaching the maximum dose for HES 10% of 30 ml kg^-1^ body weight (BW), the following, still blinded study medication was balanced crystalloid solution until a maximum of 50 ml kg^-1^ BW was reached. Thereafter, the protocol switched to open labeled balanced crystalloid solution. In analogy, we used open labeled balanced crystalloid solution after reaching a maximum dose of 50 ml kg^-1^ BW of either HES 6% or balanced crystalloid solution.

### Trial endpoints

The endpoints of this subanalysis that described the effects of the study solutions on the coagulation system were the activated partial thromboplastin time (aPTT, reference range: 26-36s), prothrombin time ratio (PTR, reference range: 70–130%), fibrinogen (reference range: 2.0–4.0g dl^-1^), platelet count (reference range: 150-400nl^-1^), von Willebrand factor antigen (vWF-Ag, reference range: 50–170%), ristocetin cofactor (vWF-RiCo, reference range: 50–150%) and factor VIII activity (reference range: 70–200%). Additionally, we measured rotational thromboelastometry (ROTEM^®^delta, Tem innovations GmbH, Munich, Germany) focusing on coagulation time (CT, reference range: 38-79s) and maximum clot firmness (MCF, reference range: EXTEM^®^ 50-72mm; FIBTEM^®^ 9-25mm) in EXTEM^®^ and FIBTEM^®^ as point-of-care testing. Furthermore, the volume of blood loss was measured by surgical suction device and the number of transfused packed red blood cells were calculated.

Blood samples were drawn into either tri-sodium-citrate-buffered or EDTA-buffered tubes (Vacutainer^®^, Becton Dickinson, Heidelberg, Germany) at three time points (1.) prior to the first application of the study medication (baseline, BL); (2.) at the end of surgery (EOS) and (3.) in the morning of the first postoperative day (POD 1) except for fibrinogen. All coagulation measurements were performed by the local laboratory immediately after blood sampling.

### Statistical analysis

After an interim analysis of the first 60 patients included in the pilot phase of the index study, a new sample size calculation was done without unblinding based on the pool standard deviation of HES solution administered. This sample size calculation resulted in a need for 753 patients in each group. In consequence, we followed the recommendation of an independent data monitoring committee (IDMC) and terminated the study after including 63 patients.

Data was analyzed descriptively; metric variables are presented as medians with 25%/75% quartiles. Categorical variables are characterized by absolute and relative frequencies. In order to model the two-way factorial design we applied linear mixed model analysis, with time and type of solution as fixed factors as well as with the patient identifier as a random factor with subsequent Tukey-type multiple comparison tests (R-packages “lme4” and “lsmeans”), which adjust for multiple testing within an endpoint. However, due to the exploratory nature of this study, we did not control for multiplicity over all endpoints simultaneously Hence, p-values are interpreted as hypothesis generating, not in a confirmatory manner. We considered a p-value < 0.05 as statistically significant. Blood loss was analyzed with pairwise Wilcoxon rank sum tests due to its skewed distribution. Transfusion requirements were only reported descriptively since few patients required transfusion.

## Results

**Fig 1:**
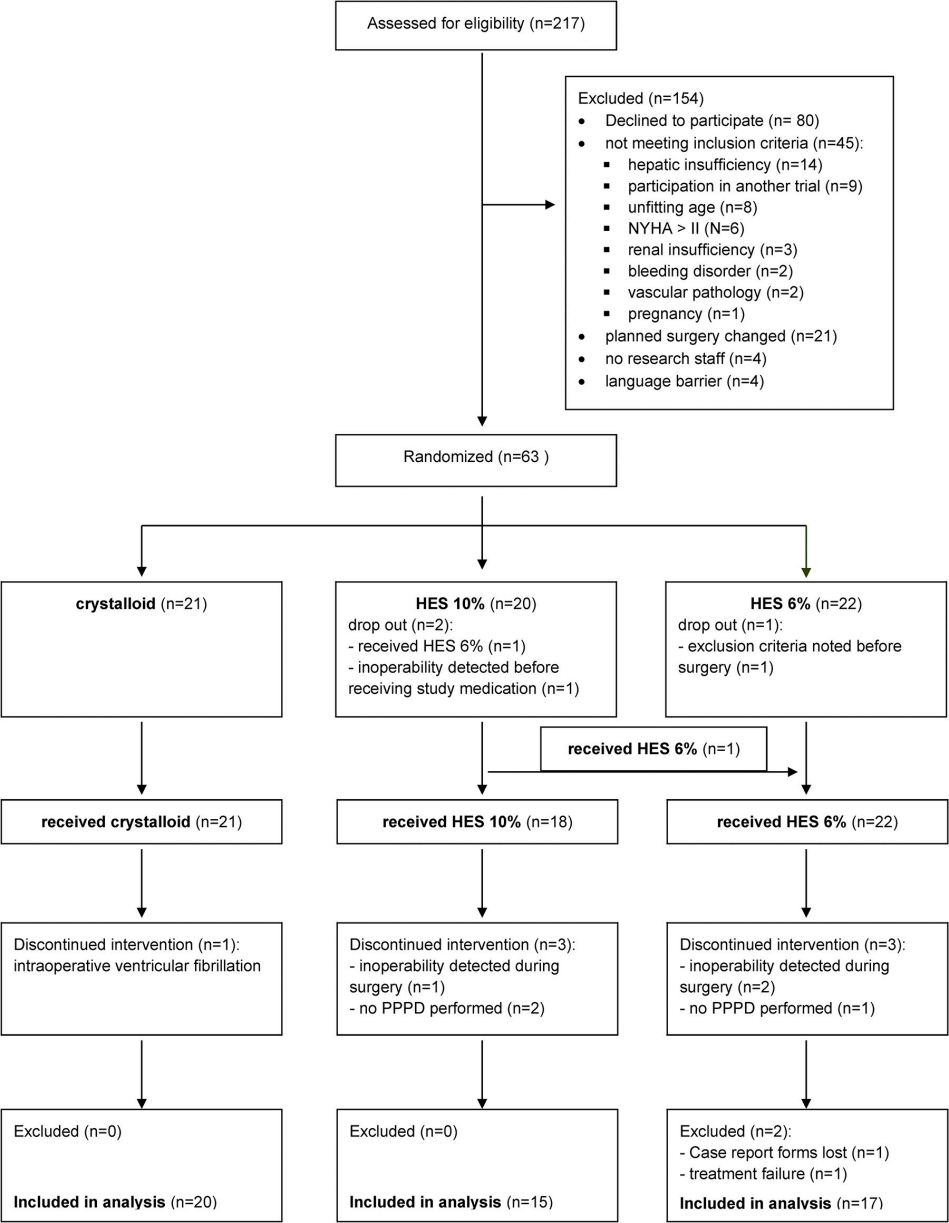
Consort flow diagram. Neither basic patient characteristics nor indication for surgery showed any significant differences between the study groups. Basic intraoperative data including volumes of study drugs and fluid requirements were not different between the groups (Table 1).

Until study termination, 217 patients were screened. 63 of them fulfilled inclusion criteria and gave their written informed consent to participate. After excluding 11 patients due to not having received study medication (n = 2), change of intraoperative procedure (n = 7), loss of reports (n = 1) or wrong application of the study medication (n = 1) (a total of 52 patients remained for statistical analysis (HES 10% (n = 15), HES 6% (n = 17) and crystalloid (n = 20) (Fig 1).

**Table 1:** Basic characteristics and intraoperative data.

	units	HES 10%	HES 6%	crystalloid
		(n = 15)	(n = 17)	(n = 20)
Age	[years]	58 [52;65]	63 [59; 72]	59 [50;68]
Body weight	[kg]	80 [72;84]	64 [61;77]	77 [67,86]
Body mass index	[kg/m^2^]	26.8 [23.2;27.8]	22.8 [20.8;25.4]	25.7 [22.7; 29.0]
ASA				
ASA I	[n; %]	0 [0%]	3 [17.6%]	1 [5.0%]
ASA II	[n; %]	14 [93.3%]	10 [58.8%]	15 [75.0%]
ASA III	[n; %]	1 [6.7%]	4 [23.5%]	4 [20.0%]
Indication for surgery				
Carcinoma of pancreatic head	[n; %]	7 [46.7%]	11 [64.7%]	11 [55.0%]
Other pancreatic tumor	[n; %]	2 [13.3%]	1 [5.9%]	2 [10.0%]
Chronic pancreatitis	[n; %]	3 [20.0%]	3 [17.6%]	6 [30.0%]
Other	[n; %]	3 [20.0%]	2 [11.8%]	1 [5.0%]
Comorbidities				
Arterial hypertension	[n; %]	6 [40.0%]	7 [41.2%]	10 [50.0%]
Coronary heart disease	[n; %]	1 [6.7%]	1 [5.9%]	1 [5.0%]
Diabetes mellitus	[n; %]	3 [20.0%]	5 [29.4%]	2 [10.0%]
**Basic intraoperative data**				
Body temperature	[°C]	36.1 [35.7;36.3]	35.9 [35.6;36.2]	36.1 [35.7;36.4]
Duration anaesthesia	[min]	419 [410; 487]	445 [400; 505]	450 [410;482]
Duration surgery	[min]	330 [288; 380]	330 [305; 400]	335 [304; 379]
Cumulative volume study medication	[ml]	2250 [2000; 2750]	2250 [1750;3000]	2500 [2062;3500]
Cumulative volume basal crystalloid	[ml]	2268 [1662; 2748]	2000 [1671; 2272]	2358 [2070; 2560]

Results are presented either as median (25% / 75% quartile) or in absolute numbers (percentage). ASA = American Society of Anesthesiologists, HES = hydroxyethyl starch

Regarding the outcomes Table 2 shows standard parameters of the coagulation system as PTR, aPTT, fibrinogen and platelet count in the intergroup analysis. We measured a decrease of the PTR for both HES groups at EOS compared to crystalloid, while the results of both HES groups were not different at EOS. An increase in the aPTT and a decrease of fibrinogen was measured only for HES 6% vs. crystalloid at EOS. On POD 1 there were no more differences for PTR and aPTT between the groups. The platelet count was not different in the intergroup comparison.

**Table 2:** Standard coagulation parameters.

		HES 10% (n = 15)	HES 6% (n = 17)	p HES 6% vs. HES 10%	Crystalloid (n = 20)	p HES 10% vs. Crystalloid	p HES 6% vs. Crystalloid
**PTR**							
PTR BL	[%]	89.0 [84.0;96.0]	89.0 [81.0;97.0]	0.99	88.5 [82.5;100]	0.81	0.89
PTR EOS	[%]	69.0 [55.0;76.0]	65.0 [57.0;72.0]	0.69	79.0 [72.5;88.0]	<0.01	<0.01
PTR POD1	[%]	64.5 [57.2;71.8]	67.0 [55.0;70.0]	0.95	67.0 [57.0; 73.5]	0.82	0.62
**aPTT**							
aPTT BL	[s]	31.7 [31.3;33.9]	33.2 [32.4;34.8]	0.80	33.4 [32.5;35.8]	0.87	0.99
aPTT EOS	[s]	36.5 [33.1;39.3]	36.9 [34.6;41.1]	0.75	31.9 [31.7;34.6]	0.19	0.03
aPTT POD1	[s]	38.9 [35.8;42.0]	39.7 [35,0;41.7]	1.00	40.2 [37.1;42.7]	0.99	1.00
**Fibrinogen**							
fibrinogen BL	[mg/dl]	338[298;378]	385 [302;442]	0.97	408 [325;458]	1.00	0.96
fibrinogen EOS	[mg/dl]	192 [163;234]	174 [163;224]	0.90	313 [248;370]	0.09	0.02
**Platelets**							
platelets BL	[/nl]	210 [184;258]	251 [200;362]	0.09	271 [171;298]	0.79	0.24
platelets EOS	[/nl]	192 [142;206]	195 [170;254]	0.62	234 [132;259]	0.60	1.00
platelets POD1	[/nl]	217 [147;264]	198 [172;247]	0.92	192 [149;228]	0.83	0.55

Results are presented as median [25%; 75% quartile]. HES = hydroxyethyl starch, PTR = prothrombin ratio, BL = baseline, EOS = end of surgery, POD1 = postoperative day 1, aPTT = activated partial thromboplastin time

In the subgroup analysis over time (Table 3), we noted decreases for PTR and fibrinogen between baseline and EOS as well as for BL to POD 1 for all study drugs. Furthermore we measured an increase in aPTT except for the crystalloid group in the comparison between BL and EOS. PTR and aPTT were significantly altered in the EOS to POD 1 comparison in the crystalloid group only. PTR decreased below the reference range on POD 1 for the crystalloid group, and at EOS and POD 1 for both HES groups, while aPTT remained within the reference range for all groups and time points.

**Table 3:** Standard coagulation parameters over time.

**PTR**	PTR BL [%]	PTR EOS [%]	PTR POD1 [%]	p BL vs EOS	p BL vs POD1	p EOS vs POD 1
HES 10% (n = 15)	89.0 [84.0;96.0]	69.0 [55.0;76.0]	64.5 [57.2;71.8]	<0.01	<0.01	0.84
HES 6% (n = 17)	89.0 [81.0;97.0]	65.0 [57.0;72.0]	67.0 [55.0;70.0]	<0.01	<0.01	0.95
Crystalloid (n = 20)	88.5 [82.5;100]	79.0 [72.5;88.0]	67.0 [57.0; 73.5]	<0.01	<0.01	<0.01
**aPTT**	aPTT BL [s]	aPTT EOS [s]	aPTT POD1 [s]			
HES 10% (n = 15)	31.7 [31.3;33.9]	36.5 [33.1;39.3]	38.9 [35.8;42.0]	0.02	<0.01	0.41
HES 6% (n = 17)	33.2 [32.4;34.8]	36.9 [34.6;41.1]	39.7 [35,0;41.7]	0.01	<0.01	0.83
Crystalloid (n = 20)	33.4 [32.5;35.8]	31.9 [31.7;34.6]	40.2 [37.1;42.7]	0.94	<0.01	<0.01
**Fibrinogen**	fibrinogen BL [mg/dl]	fibrinogen EOS [mg/dl]				
HES 10% (n = 15)	338[298;378]	192 [163;234]		<0.01		
HES 6% (n = 17)	385 [302;442]	174 [163;224]		<0.01		
crystalloid (n = 20)	408 [325;458]	313 [248;370]		0.01		
**Platelets**	platelets [/nl] BL	platelets [/nl] EOS	platelets [/nl] POD1			
HES 10% (n = 15)	210 [184;258]	192 [142;206]	217 [147;264]	0.02	0.48	0.26
HES 6% (n = 17)	251 [200;362]	195 [170;254]	198 [172;247]	<0.01	<0.01	0.84
crystalloid (n = 20)	271 [171;298]	234 [132;259]	192 [149;228]	0.09	<0.01	0.60

Results are presented as median [25%; 75% quartile]. HES = hydroxyethyl starch, PTR = prothrombin ratio, BL = baseline, EOS = end of surgery, POD1 = postoperative day 1, aPTT = activated partial thromboplastin time

Platelet count showed in the BL to EOS comparison a decrease for both HES groups, while in the BL to POD 1 comparison the decrease was significant for the HES 6% and the crystalloid group. At any time point it remained within the reference range for all solutions.

**Fig 2:**
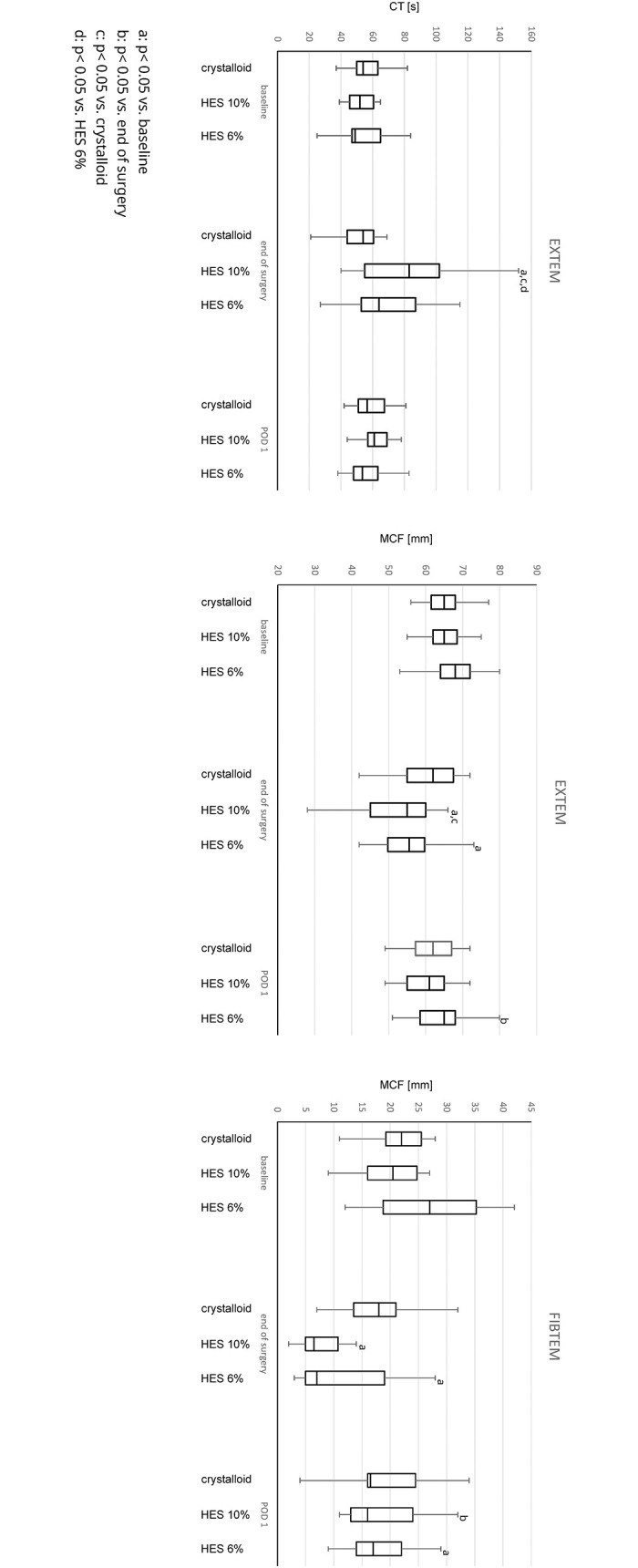
ROTEM. CT = clotting time, MCF = maximum clot firmness, HES = hydroxyethyl starch, a: p<0.05 vs. baseline, b: p<0.05 vs. end of surgery, c: p< 0.05 vs. crystalloid, d: p< 0.05 vs. HES 6%.

Fig 2 shows the results of the ROTEM^®^ measurements as CT and MCF for the EXTEM^®^ and MCF for the FIBTEM^®^ assays between the study groups and over time:

The CT EXTEM^®^ was significantly prolonged for the HES 10% group compared to the other groups at EOS and in the baseline to EOS comparison over time. This effect did not last on POD 1 in the HES 10% group. Crystalloid and HES 6% volume replacement exerted no changes on the CT EXTEM^®^ over time.

The prolongation in CT EXTEM^®^ for HES 10% exceeded the reference range at EOS, while all other values remained in the reference range at all time points.

In the intergroup comparison of the MCF EXTEM^®^ there was only a decrease at EOS for the HES 10% group compared to crystalloids. The MCF EXTEM^®^ showed significant decreases in both HES groups in the baseline to EOS comparison and a significant increase for the HES 6% group in the EOS to POD 1 comparison. On POD 1 the MCF EXTEM^®^ was no more different in the baseline to POD 1 comparison in all groups. No significant changes were noted for the crystalloid group.

For the MCF FIBTEM^®^ significant decreases were measured for both HES groups for the comparison of baseline to the EOS time point, which was not significant compared to crystalloid at the end of surgery. On POD 1 the MCF FIBTEM^®^ increased for all solutions. This increase was significant for HES 10% for the EOS to POD 1 comparison, but not for crystalloid or HES 6%. In the HES 6% group the MCF FIBTEM^®^ remained significantly smaller on POD 1 compared to baseline, while the MCF returned close to baseline values in the crystalloid and HES 10% group.

For the MCF EXTEM^®^ all values remained in the reference range on all time points, while the MCF FIBTEM^®^ decreased in both HES groups under the reference range at EOS.

Tables 4 and 5 describe the results of the von Willebrand parameters: in the intergroup analysis (Table 4) significantly decreased activities of vWF-RiCo were measured for both HES groups at the EOS time point compared to crystalloids as well as a significantly decrease of vWF-Ag and factor VIII activities for HES 6% versus crystalloids. On POD 1 all parameters were no more different between the groups.

**Table 4:** Von Willebrand parameters.

		HES 10% (n = 15)	HES 6% (n = 17)	p HES 6 vs HES 10	Crystalloid (n = 20)	p HES 10% vs crystalloid	p HES 6% vs. Crystalloid
**vWF-Ag**							
vWF-Ag BL	[%]	191 [151;226]	149 [126;204]	0.91	147 [134;208]	0.97	0.98
VWF-Ag EOS	[%]	140 [99.8;176]	123 [105;140]	0.92	176 [150;239]	0.06	0.02
VWF-Ag POD1	[%]	262 [218;324]	262 [208;305]	0.83	279 [250;412]	0.50	0.19
**vWF-RiCo**							
VWF-RiCo BL	[%]	186 [154;200]	144 [127;200]	1.00	170 [147;190]	0.99	0.98
VWF-RiCo EOS	[%]	158 [122;192]	143 [122;174]	0.97	225 [157;270]	0.03	0.01
VWF-RiCo POD1	[%]	320 [244;413]	301 [239;355]	0.51	334 [241;386]	0.98	0.36
**Factor VIII**							
factor VIII BL	[%]	193 [152;212]	165 [134;218]	0.65	185 [140;227]	0.72	0.99
factor VIII EOS	[%]	112 [83.0;163]	117 [90.0;139]	0.67	202 [151;268]	0.05	<0.01
factor VIII POD1	[%]	246 [224;270]	234 [221;261]	0.55	221 [190;339]	0.88	0.82

Results are presented as median [25%; 75% quartile]. HES = hydroxyethyl starch, vWF-Ag = von Willebrand factor antigen, BL = baseline, EOS = end of surgery, POD1 = postoperative day 1, vWF-RiCo = von Willebrand factor ristocetin cofactor

**Table 5:** Von Willebrand parameters over time.

vWF-Ag	vWF-Ag [%] BL	vWF-Ag [%] EOS	vWF-Ag [%] POD1	p BL vs EOS	p BL vs POD1	p EOS vs POD1
HES 10% (n = 15)	191 [151;226]	140 [99.8;176]	262 [218;324]	0.01	<0.01	<0.01
HES 6% (n = 17)	149 [126;204]	123 [105;140]	262 [208;305]	<0.01	<0.01	<0.01
Crystalloid (n = 20)	147 [134;208]	176 [150;239]	279 [250;412]	0.12	<0.01	<0.01
**vWF-RiCo**	vWF-RiCo [%] BL	vWF-RiCo [%] EOS	vWF-RiCo [%] POD1	p BL vs EOS	p BL vs POD1	p EOS vs POD1
HES 10% (n = 15)	186 [154;200]	158 [122;192]	320 [244;413]	0.75	<0.01	<0.01
HES 6% (n = 17)	144 [127;200]	143 [122;174]	301 [239;355]	0.56	<0.01	<0.01
Crystalloid (n = 20)	170 [147;190]	225 [157;270]	334 [241;386]	0.02	<0.01	<0.01
**Factor VIII**	factor VIII [%] BL	factor VIII [%] EOS	factor VIII [%] POD1	p BL vs EOS	p BL vs POD1	p EOS vs POD1
HES 10% (n = 15)	193 [152;212]	112 [83.0;163]	246 [224;270]	<0.01	0.04	<0.01
HES 6% (n = 17)	165 [134;218]	117 [90.0;139]	234 [221;261]	<0.01	0.05	<0.01
Crystalloid (n = 20)	185 [140;227]	202 [151;268]	221 [190;339]	0.29	<0.01	0.14

Results are presented as median [25%; 75% quartile]. HES = hydroxyethyl starch, vWF-Ag = von Willebrand factor antigen, BL = baseline, EOS = end of surgery, POD1 = postoperative day 1, vWF-RiCo = von Willebrand factor ristocetin cofactor

In the analysis over time (Table 5) the crystalloid group and both HES groups showed a significant increase in the activity of vWF-AG and vWF-RiCo in the baseline versus POD 1 and EOS versus POD 1 analysis. FVIII activity showed a different pattern with a significant decrease from baseline to EOS and a significant increase in the EOS to POD 1 comparison for both HES groups while factor VIII activity showed a significant increase from BL to POD 1 in the crystalloid group.

Intraoperative blood loss was not significantly different between the groups (Table 6).

**Table 6:** Blood loss and transfusion requirements.

	HES 10%	HES 6%	p HES 6% vs. HES 10%	crystalloids	p HES 10% vs. Crystalloid	p HES 6% vs. Crystalloid
	(n = 15)	N (= 17)		(n = 20)		
Blood loss [ml]	900 [650; 1650]	750 [500;1500]	0.55	750 [400;1000]	0.18	0.54
Patients receiving PRBC intraoperative [n; %]	6 [40%]	6 [35.29%]		4 [20%]		
0 PRBC	9 [60%]	11 [64.71%]		16 [80%]		
1 PRBC	2 [13.33%]	2 [11.76%]		0 [0%]		
2 PRBC	2 [13.33%]	1 [5.88%]		2 [10%]		
3 PRBC	1 [6.67%]	1 [5.88%]		2 [10%]		
≥ 4 PRBC	1 [6.67%]	2 [11.76%]		0 [0%]		
Patients receiving PRBC postoperative [n; %]	2 [13.33%]	2 [11.76%]		1 [5%]		
0 PRBC	12 [80%]	15 [88.24%]		19 [95%]		
1 PRBC	2 [13.33%]	1 [5,88%]		1 [5%]		
2 PRBC	1 [6.67%]	0 [0%]		0 [0%]		
3 PRBC	0 [0%]	1 [5.88%]		0 [0%]		
Patients receiving FFP intraoperative [n; %]	4 [26.67%]	4 [23.53%]		6 [30%]		
0 FFP	11 [73.33%]	13 [76.47%]		14 [70%]		
1 FFP	1 [6.67%]	0 [0%]		1 [5%]		
2 FFP	1 [6.67%]	0 [0%]		1 [5%]		
3 FFP	1 [6,67%]	1 [5.88%]		1 [5%]		
≥ 4 FFP	1 [6.67%]	3 [17.65%]		3 [15%]		
Patients receiving FFP postoperative [n; %]	1 [6.67%]	2 [11.76%]		1 [5%]		
0 FFP	14 [93.33%]	15 [88.24%]		19 [95%]		
1 FFP	0 [0%]	0 [0%]		0 [0%]		
2 FFP	1 [6.67%]	1 [5.88%]		1 [5%]		
3 FFP	0 [0%]	1 [5.88%]		0 [0%]		

Results are presented as median [25%; 75% quartile] for blood loss and in absolute numbers and percentage for transfusions. PRBC = packed red blood cells, HES = hydroxyethyl starch, FFP = fresh frozen plasma

Intraoperative only 4 patients received packed red blood cells (PBRC) in the crystalloid group, while in both HES groups 6 patients received PRBC. Of these patients only 1 patient in the crystalloid group and 2 patients in each HES group received PRBC postoperative. For the transfusion of fresh frozen plasma (FFP) there were 6 patients in the crystalloid group and 4 patients in each HES group who received FFP during surgery. Postoperative only 1 patient received FFP in the HES 10% and in the crystalloid group and 2 patients in the HES 6% group.

## Discussion

The main findings of the presented study are that (1) significant impairments in certain parameters of the coagulation laboratory after major abdominal surgery were found in patients receiving balanced 6% and 10% HES solutions used in a hemodynamic optimization protocol and dosed until the maximum daily dose was reached as compared to crystalloids; (2) that these changes either normalized or remained stable on POD 1 with the exception of the MCF in the HES 6% group which remained significantly reduced compared to baseline and EOS; and (3) that, intraoperative blood loss was not significantly different between the study groups.

In recent years the effect of different volume replacement solutions on the plasmatic coagulation system focused on in vitro studies using point of care devices (thromboelastometry or thromboelastography) or in vitro studies [20, 21]. The drawbacks of these studies are the missing or reduced effects of the biological environment (missing endothelial response to trauma, activation of the plasmatic coagulation system, increase in coagulation factor activities and platelet numbers after surgery) [22] and the lack of clinical consequences of hemodilution, e.g. blood loss and transfusion requirements, so that the discussion of our results mainly refers to clinical studies.

A meta-analysis of in vivo studies investigated the effect of HES based volume replacement on clinical outcomes [23]: for non-cardiac surgery there was a more pronounced reduction in maximum amplitude as measured by thromboelastography using HES solutions, which corresponds well with our findings for the EOS time point. However, a significantly higher blood loss and transfusion requirement after receiving HES-based solutions (including low molecular weight HES) versus crystalloid in non-cardiac surgery was reported. The difference to our results may be explained by using a hemodynamic optimization protocol for the study solutions and packed red blood cells and fresh frozen plasma to prevent an uncritical volume and blood product replacement.

An earlier prospective, randomized, double-blinded study investigated the effects of acute hypervolemic fluid infusion (AFHI) with 30ml/kg HES 6% (130 kD) and Ringer’s Lactate during gastrectomy [24]. Similar to our findings the authors described significant changes over time for both groups and for the PT after AFHI, but without difference in the intergroup comparison after 4 hours. Thromboelastography showed a significantly reduced clot firmness and prolonged reaction time for HES after AFHI, but did not compare differences between the groups. Different to our study, this study did not measure differences in vWF-Ag levels and a decreased factor VIII activity. Despite these changes, the intraoperative blood loss was–similar to our study—not different between the study groups.

However, due to the short observation period this study did not monitor the further course of the coagulation parameters, e.g. increase in coagulation parameters on the first postoperative day and the postoperative blood loss.

Another prospective, randomized clinical study of 66 orthopedic patients undergoing greater spine surgery investigated the effects of volume replacement with HES 6% (130 kD) versus Ringer’s Lactate solution [25].

Similar to our findings the authors showed a significant prolongation for the aPTT in the intergroup comparison at 3 hours after start of surgery. This difference persisted for 6 hours after start of surgery, which is confirmed by our results at the EOS time point, and supports a transient effect of HES solutions on coagulation parameters.

The results of their thromboelastometric analysis are, in part, in line with our results showing no difference between HES 6% and crystalloid on the CT EXTEM^®^. The decrease in MCF FIBTEM^®^ and factor VIII activity in both HES groups at the end of surgery was confirmed by Mittermayr for the time point 3 hours after start of surgery.

However, our results describe a significant decrease in Ristocetin-cofactor activity, MCF FIBTEM^®^ and factor VIII activity, that remained either unchanged (or not measured) in the work of Mittermayr [25]. This may be explained by a smaller infusion volume of HES as compared to our study. The calculated blood losses between the groups were not different in this study, however the HES group received more red cell transfusion than the crystalloid group. This finding is not supported by our results, which may be explained by the avoidance of unnecessary volume replacement by the use of a hemodynamic optimization protocol in our study. Despite a recent published meta-analysis also showed a higher need in intraoperative blood transfusions when using HES solutions for volume replacement [26].

The clinical data on the impact of 10% HES on the coagulation system is still rare. Shin et al. compared in a blinded randomized clinical trial for patients undergoing hip arthroplasty three different HES solutions (Pentastarch 10% 260/0.45; Tetrastarch 6% 130/0.4 either in 0,9% saline or balanced electrolyte solution) [27]. They showed a reduced platelet count directly after as well as on the second day after surgery. All groups showed a reduced PTR, but only the 10% pentastarch and the saline based 6% HES solution showed a prolonged aPTT after surgery. Also MCF FIBTEM^®^ and MCF EXTEM^®^ were reduced at the end of surgery in all groups. For these parameters the changes were significantly more pronounced in the HES 10% group. In contrast, our results do not confirm a difference between balanced HES 6% and HES 10% solution at the EOS time point. Moreover, our results suggest a transient impact of starch solutions on coagulation parameters and clot strength at the end of surgery, as the coagulopathic effect of the investigated HES solutions remained stable on the first postoperative day and was not associated with significantly different blood losses or transfusions requirements.

With regard to the effect of HES on the von Willebrand-system and factor VIII, the triple-blinded randomized clinical trial by Arellano et al. showed a decrease in vWF-Ag and factor VIII-activity after infusion of up to 45ml/kg of 10% HES (264/0.45) against 5% albumin solution over time [28]. Our data confirm a decrease of vWF-Ag, vWF-RiCo and factor VIII-activity at the end of surgery, that was no more different on the first postoperative day, which suggest either a transient impact of the HES solutions on these parameters or a postoperative acute phase reaction with release of FVIII and von Willebrand factor from the endothelium [29]. Unfortunately no postoperative coagulation data are reported by Arellano et al. so that the time course of the coagulopathic effect of HES cannot be assessed.

## Limitations

The major limitation of this analysis is the small number of participants due to the early stop of enrollment for futility. Out of this reason the results should be regarded as exploratory and hypothesis-generating only, but may serve as a basis for larger trials in the future. Furthermore, our data from surgery of the pancreatic head may not be generalizable to other major abdominal surgeries with less trauma and blood loss, but may well work as a model for major surgeries with higher blood loss and transfusion requirements.

Moreover, the volume replacement following the hemodynamic optimization algorithm was only performed during surgery and not continued in the ICU. However, the attending intensivists of the postoperative ICU were aware of the study enrollment and the aim of the study but not of study group allocation. The measurements of coagulation parameters were not continued after POD 1 and did not comprise parameters of the endothelial activation, so that a prolonged impact of the study solutions on the coagulation system and the endothelium could not be assessed.

The strengths of our study are that we used a double-blind prospective setting to avoid confounding, used two different HES concentrations and a crystalloid control group and applied a goal-directed optimization algorithm to prevent uncritical infusion of HES solutions in clinical practice in a fairly homogenous group of patients and a standardized surgical procedure. Moreover, we used a variety of coagulation parameters that included parameters of the standard coagulation laboratory, of the von Willebrand-system and thromboelastometric analyses to provide a broad assessment of the impact of different HES solutions on the coagulation system.

## Conclusion

Our data suggest that volume replacement with low molecular 6% and 10% HES for major pancreatic surgery stronger deteriorates the standard and point of care parameters of the coagulation system compared to crystalloid solutions. This effect was statistically significant at the end of surgery, but no more on the first postoperative day. This may be interpreted as a transient effect of low-molecular 6% and 10% HES preparations on the coagulation system that did not result in increased blood loss or transfusion requirements.

## Supporting information

S1 Checklist Reporting checklist for randomised trial. (DOCX)

S1 Data (XLSX)

S1 File (PDF)
