# Supplementary material for: In vivo effects of balanced, low molecular 6% and 10% hydroxyethyl starch compared with crystalloid volume replacement on the coagulation system in major pancreatic surgery—a sub-analysis of a prospective double-blinded, randomized controlled trial
Source: PLoS One. 2024 Jul 11;19(7):e0303165. doi: 10.1371/journal.pone.0303165 (PMC11239059; doi:10.1371/journal.pone.0303165)
Supplement: S1 File — (PDF) [file pone.0303165.s003.pdf]

CLINICAL STUDY PROTOCOL

PROSPECTIVE, CONTROLLED, DOUBLE-BLIND, RANDOMIZED MULTICENTRIC STUDY
ON THE EFFICACY AND SAFETY OF
A TARGET CONTROLLED PLASMA VOLUME REPLACEMENT THERAPY WITH
A HYPER-ONCOTIC BALANCED HES 130/0.42 SOLUTION VS
AN ISO-ONCOTIC BALANCED HES 130/0.42 SOLUTION COMPARED TO
A BALANCED ELECTROLYTE SOLUTION
IN ELECTIVE SURGERY OF THE PANCREATIC HEAD

STUDY IDENTIFICATION NO.: HC-G-H-0803
EUDRACT NO.: 2008-004175-22

HOSPITAL CARE
B. BRAUN MELSUNGEN AG
CARL-BRAUN-STR. 1
34212 MELSUNGEN
GERMANY

consolidated version<br>dated	03<br>2010-03-08
based on version<br>dated	01<br>2008-11-11
incorporating Amendment no<br>dated	01<br>2009-06-15
incorporation amendment no<br>dated	02<br>2010-03-08
incorporation amendment no<br>dated	03<br>2010-12-17
and incorporating amendment no<br>dated	04<br>2011-09-05

This protocol has been written in accordance with current ICH-GCP guidelines

TABLE OF CONTENTS

1 RESPONSIBILITIES AND ADDRESSES 5
2 ABBREVIATIONS AND DEFINITIONS 9
3 PROTOCOL SUMMARY / SYNOPSIS 12
4 INTRODUCTION 26
5 STUDY RATIONALE 26
6 RISK-BENEFIT-ASSESSMENT 27
7 STUDY OBJECTIVES 27
7.1 PRIMARY OBJECTIVE 27
7.1.1 Primary Variable 27
7.2 SECONDARY OBJECTIVES 27
8 STUDY DESIGN 27
9 SELECTION AND WITHDRAWAL OF PATIENTS 29
9.1 INFORMED CONSENT 29
9.2 PATIENT INCLUSION CRITERIA 29
9.3 PATIENT EXCLUSION CRITERIA 29
9.4 STOPPING AND DISCONTINUATION CRITERIA 30
9.4.1 Discontinuation Criteria Related to the Study 30
9.4.2 Discontinuation Criteria Related to the Study Site 30
9.4.3 Discontinuation Criteria related to the Patient 31
9.5 RANDOMISATION, BLINDING AND UNBLINDING 32
10 INVESTIGATIONAL PRODUCTS 33
10.1 NAME AND DESCRIPTION OF THE INVESTIGATIONAL PRODUCT(S) 33
10.1.1 Qualitative and Quantitative Composition 33
10.1.2 Pharmaceutical Form 35
10.1.3 Nature and Content of Container(s) 35
10.2 POSOLOGY AND METHOD OF ADMINISTRATION 35
10.2.1 Dosage 35
10.2.2 Method of Administration 36
10.2.3 Duration of treatment 36
10.3 LABELLING 36
10.4 PACKAGING 37
10.4.1 Storage 37
10.4.2 Investigational Product Accountability 37
10.5 DESTRUCTION / RETRIEVAL OF SURPLUS INVESTIGATIONAL PRODUCTS 38
11 CONCOMITANT THERAPY 38
12 DEFINITION OF THE PRIMARY AND SECONDARY VARIABLES 39
12.1 PRIMARY VARIABLE 39
12.2 SECONDARY VARIABLES - SAFETY 39
12.3 SECONDARY VARIABLES - EFFICACY 42
12.4 OTHER VARIABLES 44
12.5 SOURCE DOCUMENTS 44
13 ASSESSING AND REPORTING OF ADVERSE EVENTS 45
13.1 DEFINITIONS 45
13.1.1 Adverse Events 45
13.1.2 Adverse Reaction 45
13.1.3 Unexpected Adverse Reactions 45
13.1.4 Serious Adverse Event (SAE) or Serious Adverse Reaction (SAR) 46

13.1.5 Adverse Event Intensity *46*
13.1.6 Adverse Event Causality *46*
13.2 RECORDING AND REPORTING ADVERSE EVENTS AND ADVERSE REACTIONS 47
13.2.1 Recording *47*
13.2.2 Reporting of Serious Adverse Events and Unexpected Adverse Reactions *48*
13.3 ADVERSE EVENT FOLLOW-UP PROCEDURES 48
13.4 POTENTIAL RISKS AND POTENTIAL ADVERSE EVENTS 48
13.5 PREGNANCIES 49

14 VISIT SCHEDULE 49

14.1 PROCEDURES AT EACH VISIT 49
14.2 TABULAR OVERVIEW 54
14.3 ASSESSMENT OF COMPLIANCE 57
14.4 PRECAUTIONARY MEASURES 57

15 DURATION OF STUDY AND STUDY SCHEDULE 58

15.1 DURATION OF STUDY PER PATIENT 58
15.2 STUDY SCHEDULE: DURATION OF WHOLE STUDY 58

16 STATISTICS 58

16.1 STATISTICAL METHODS 58
16.2 LEVEL OF SIGNIFICANCE AND POWER 59
16.3 STATISTICAL HYPOTHESES 59
16.4 SAMPLE SIZE 59
16.5 DATA HANDLING 60
16.6 INTERIM ANALYSIS 60
16.7 CRITERIA FOR THE TERMINATION OF THE STUDY 61
16.8 PATIENT SELECTION FOR ANALYSES 61

17 SOURCE DATA AND SOURCE DOCUMENTS 61

17.1 DEFINITIONS 61
17.1.1 Source Documents *61*
17.1.2 Source Data *61*
17.1.3 Direct Access *61*
17.2 PERMISSION OF ACCESS 62

18 QUALITY CONTROL AND QUALITY ASSURANCE 62

18.1 QUALITY CONTROL 62
18.1.1 Definition *62*
18.1.2 Study Monitoring *62*
18.2 QUALITY ASSURANCE 62
18.2.1 Definition *62*
18.2.2 Audit *62*
18.2.3 Inspection *63*

19 ETHICAL AND LEGAL CONSIDERATIONS 63

19.1 COMMITTEES AND BOARDS 63
19.1.1 Independent Ethics Committee (IEC) *63*
19.1.2 Institutional Review Board (IRB) *63*
19.1.3 Drug Safety Monitoring Board (DSMB) *63*
19.2 CONDUCT OF STUDY AND ETHICAL CONSIDERATIONS 64
19.3 RESPONSIBILITIES 64
19.4 GENERAL REPORTING OBLIGATION 64
19.5 FINANCING AND INSURANCE 64
19.6 PERSONAL DATA AND DATA PROTECTION 65
19.7 MODIFICATION OF PROTOCOL 65
19.8 INVESTIGATOR'S BROCHURE/SUMMARY OF PRODUCT CHARACTERISTICS 65
19.9 COMPLETION OF CASE REPORT FORMS 65
19.10 ARCHIVING 66
19.11 CONFIDENTIALITY 66

20 FINAL REPORT AND PUBLICATION POLICY 66

21 REFERENCES 68

22 SIGNATURES 70

23 APPENDICES 71

1 Declaration of Helsinki (version 1996)

2 Patient Information Sheet and Informed Consent Form (version 02)

3 Screening Log

4 Enrolment Log

5 Randomisation Sheet

6 Dosing algorithm (v02)

7 Inventory Form (accountability)

8 Scores

9 Serious Adverse Event Form

1 Responsibilities and addresses

Coordinating Investigator

„Leiter der klinischen Prüfung“

Principal Investigator Centre1
„Hauptprüfer“ Centre 1

Investigator(s) Centre 1
„Prüfer“ Centre 1

Principal Investigator Centre 2
„Hauptprüfer“ Centre 2

Investigator(s) Centre 2
„Prüfer“ Centre 2

Principal Investigator Centre 3
„Hauptprüfer“ Centre 3

Investigator(s) Centre 3
„Prüfer“ Centre 3

Sponsor

Sponsor’s representative

Medical representative of the Sponsor

Study co-ordinator

Statistician (Planning)

Statistician (Evaluation)

Randomisation

SAE contact details

CRO/Monitor

Laboratory

2 ABBREVIATIONS AND DEFINITIONS

AE	Adverse event
ACE-I	Angiotensin converting enzyme-inhibitor
ANOVA	Analysis of variance
APACHE-score	Acute physiology and chronic health evaluation-score
aPTT	activated partial thromboplastin time
APS	American pain society
ASA	American society of anesthesiologists
AR	Adverse reaction
AT	Antithrombin
BE	Base excess
BGA	Blood gas analysis
BMI	Body mass index
BNP, NT-Pro-BNP	Brain natriuretic peptide, N-terminal pro brain natriuretic peptide
BUN	Blood urea nitrogen
Ca	Calcium
CFT	Clot formation time
CI	Cardiac Index
Cl	Chloride
CPAP	Continuous positive airway pressure
CRF	Case report form
CRO	Contract research organisation
CRP	C-reactive protein
CT	Clotting time
CTM	Clinical trial material
CVP	Central venous blood pressure
d	Day
DAP	Diastolic arterial blood pressure
DBP	Diastolic blood pressure
D-DRG	German diagnosis related group
DOB	Date of birth
EC	Erythrocyte concentrate
ECG	Electrocardiography
EF	Ejection fraction
EVLWI	Extravascular lung water index
FVIII:C	Factor VIII plasma activity
FAS	Full analysis set

FFP	Fresh frozen plasma
GCP	Good clinical practice
GMP	Good manufacturing practise
h	Hour
Hb	Haemoglobin
HbA1c	Haemoglobin A1c
Hct	Haematocrit
HES	Hydroxyethyl starch
HR	Heart rate
HRQoL	Health related quality of life
ICH	International Conference on Harmonisation
ICU	Intensive care unit
IEC	Independent ethics committee
IL	Interleukin
IMCU	Intermediate care unit
INR	International normalized ratio for prothrombin time
IRB	Institutional review board
ITBVI	Intrathoracic blood volume index
ITT	Intent-to-treat
K	Kalium, potassium
kg	kilogram
MAP	Mean arterial blood pressure
α1-MG	α1-Mikroglobulin
MELD-score	Model of end stage liver system-score
min	Minute
Na	Natrium
NA	Not applicable
β-NAG	beta-N-acetylglucosaminidase
NYHA	New York heart association
PC	Platelet concentrate
PCT	plasma clotting time
PCV	Packed cell volume
pCO2	Partial pressure of carbon dioxide
pO2	Partial pressure of oxygen
PONV	Postoperative nausea and vomitting
PP	Per protocol
PPPD	Pylorus preserving pancreatic-duodenectomy
PT	Prothrombin time

PTT	Partial thromboplastin time, plasma thrombin time
RBC	Red blood cell count
SAE	Serious adverse event
SaO2	Arterial oxygen saturation
SAP	Systolic arterial blood pressure
SAPS	Simplified acute physiology score
SBP	Systolic blood pressure
SIRS	Systemic inflammatory response syndrome
SmPC	Summary of product characteristics
StAP	Statistical analyses plan
SOFA-score	Sequential organ failure assessment score
SOP	Standard operating procedure
SV	Stroke volume
SVR	Systemic vascular resistance
TEG	Thrombelastography
VAS	Visual analogue scale
VCAS	Valid case analysis set
vWF	von Willebrand factor
vWF-Ag	von Willebrand factor-antigen
vWF-RiCo	von Willebrand factor ristocetin co-factor
WBC	White blood cells

3 PROTOCOL SUMMARY / SYNOPSIS

Title of Study	PROSPECTIVE, CONTROLLED, DOUBLE-BLIND, RANDOMIZED MULTICENTRIC STUDY ON THE EFFICACY AND SAFETY OF TARGET CONTROLLED VOLUME REPLACEMENT THERAPY WITH A HYPERONCOTIC BALANCED HES 130/0.42 SOLUTION VS AN ISOONCOTIC BALANCED HES 130/0.42 SOLUTION COMPARED TO A BALANCED ELECTROLYTE SOLUTION IN ELECTIVE SURGERY OF THE PANCREATIC HEAD
Investigational Products	Investigational Test Product:<br>Tetraspan 10% (hyper-oncotic balanced HES 130/0.42 solution)<br>Investigational Reference Product:<br>Tetraspan® 6% (iso-oncotic balanced HES 130/0.42 solution)<br>Descriptive control:<br>Sterofundin ISO (balanced electrolyte solution)<br>A treatment group receiving solely a crystalloid/electrolyte volume replacement is included in the study as in various hospitals and countries respectively this treatment is still a standard in surgery including pancreatic surgery. As crystalloid solutions, however, display another volume effect*, than colloidal solutions (*thus leading to a distinct higher requirement of volume which could be easily recognised even when blinded) this treatment group will only serve as a descriptive control.
Phase	IV
Study Design	Prospective, controlled, randomized, double-blind, bi-(multi-)centric study performed in three parallel groups
Number of Sites & Countries	multicentric, Germany
Sample Size	Estimated: 228. The study will be performed in 2 phases:<br>The study will begin with an internal pilot phase including 60 patients. At the end of this pilot phase, the pooled variance with respect to the primary variables will be estimated without unblinding and will be used for confirmation respectively recalculation of the initially estimated sample size. Interim analysis will also serve for deciding of premature termination of the study for futility.<br>For the comparison of the defined groups at end of study, the error of the 1st kind α will be strictly adhered to with this procedure (Kieser *et al.*, 2000 and 2003).
Indication	Intraoperative plasma volume replacement in planned elective surgery of the pancreatic head (pylorus preserving pancreatic-duodenectomy, PPPD)

Primary Objective	Investigation on efficacy of target controlled fluid therapy with a hyper-oncotic balanced HES 130/0.42 solution compared to an iso-oncotic HES 130/0.42 solution in patients undergoing elective surgery of the pancreatic head. A third group receiving a balanced electrolyte solution (without colloidal volume replacement) will serve as a control for descriptive analysis.
Primary Variable	A multiple primary endpoint is chosen:<br>First endpoint: Intraoperatively required amount of HES (10%, 6%) (ml)<br>Second endpoint: Time until fully on oral (solid) diet (days)
Secondary Objectives	Investigation of safety and secondary efficacy parameters of the balanced HES 130/0.42 solutions and of the balanced electrolyte solution.
Secondary Variables	Safety:<br>▪ Haemodynamics<br>o SAP, DAP, MAP, HR, CVP – *measurements: baseline (after anaesthesia), intraoperatively every 15 min, end of surgery,on admission to ICU and on ICU* every 6 hours*<br>o CI, SV, SVR using oesophageal Doppler and PiCCO – *measurements: baseline, intraoperatively following dosing algorithm (i.e. in 15 min intervals when no volume optimisation, or 15 minutes after new optimised stroke volume),, end of surgery. PiCCO also on admission to ICU, 6 hours after admission, on ICU* from post-op day +1 on 12-hourly*<br>o cumulative (*per hour*) dose of norepinephrine (mcg/h)<br>o intrathoracic blood volume index (ITBVI) and extravascular lung water index (EVLWI) using PICCO – *measurements: baseline, intraoperatively every 60 min after start of surgery, end of surgery, on admission to ICU, 6 hours after admission, on ICU* from post-op day +1 on 12-hourly*<br>▪ blood gas analysis (pCO2, pO2, HCO3, SaO2) – *measurements: baseline, intraoperatively every 60 min after start of surgery and at the end of surgery (arterial blood sample), on admission to ICU, 6 hours after admission, 1st postoperative day every 6 hours, on ICU* from post-op day +2 until post-op day +5 once daily*<br>▪ acid-base status (pH, base excess, bicarbonate, lactate) – *measurements: baseline, intraoperatively every 60 min after start of surgery, at the end of surgery, on admission to ICU, 6 hours after admission, 1st operative day every 6 hours, on ICU* from post-op day +2 until post-op day +5 once daily*<br>▪ electrolytes (sodium, potassium, ionized calcium, chloride) – *measurements: baseline, intraoperatively every 60 min after start of surgery, at the end of surgery, on admission to ICU, 6 hours after admission, 1st*

	postoperative day every 6 hours, on ICU from post-op day +2 until post-op day +5 once daily*<br>▪ Cardiac function: Troponin or high-sensitivity Troponin, NT-Pro-BNP – *measurements: baseline, end of surgery, 1st postoperative day morning*<br>▪ inflammatory reaction<br>o facultative: IL6, IL 10, IL6/IL10-ratio – *measurements: baseline, end of surgery, 1st postoperative day in the morning*<br>o PCT, CRP *baseline, 1st postoperative day in the morning*<br>o SIRS-criteria (body temperature, leucocytes (WBC), HR, pCO2)*, 1st postoperative day in the morning, on ICU* once daily (morning)*<br>▪ Renal function<br>o diuresis/urinary output *during surgery (cumulative every 60 minutes)*, *1st postoperative day in the morning, on ICU* (urine 24 hour collection period is from morning until next morning about 6 o'clock)*<br>o Serum: creatinine, BUN (calculated from serum urea which will be measured)<br>Urine: α1-Mikroglobulin, ß-NAG, creatinine<br>*measurements: baseline, on ICU* once daily, serum creatinine also on peripheral ward on post-operative days 3 and 7*<br>o Serum: HbA1c *baseline*<br>▪ Haemostasis<br>o PT, aPTT, Fibrinogen,<br>o vWF-Ag, vWF-Ristocetin-Cofactor (vWF-RiCo),<br>o FVIII:c,<br>o CT, CFT, MCF ExTEM and FibTEM<br>*measurements: baseline, intraoperatively 1h after start* (ROTEM only)*, at the end of surgery, 1st operative day in the morning* (PT, aPTT, vWF-AG, vWF-RiCo, ROTEM)<br>▪ Circulating blood volume (mLs): Calculation of according to the following formulas:<br>o males: ((0.3669 x height (m) + 0.03219 x weight (kg) + 0.6041) x 1000<br>o females: ((0.3061 x height (m) + 0.03308 x weight (kg) + 0.1833) x 1000<br>(Nadler S, Surgery 1962, Mercuriali F, Curr Med Res Opin 1996).<br>*baseline, 1st postoperative day (if feasible)*<br>▪ Requirements of blood products (EC, FFP, PC, plasma derived coagulation factor concentrates) *during surgery*, *intraoperative* blood loss (suction volume minus rinsing fluids only)<br>▪ PONV (postoperative nausea and vomiting) *on ICU* and until post-op day 5*<br>▪ Adverse Events – *from start of administration of Investigational product continuously until postoperative day 5 (serious adverse events which are not yet resolved on POD 5 will be followed until study end).*

	* if not otherwise mentioned measurements of secondary variables on ICU will be performed until fit-for-discharge from ICU criteria are fulfilled or until postoperative day 5 (including), whatever occurs first.<br><br>Efficacy:<br>▪ Haemodynamics<br>o SAP, DAP, MAP, HR, CVP, CI, SV, SVR<br>o cumulative dose of norepinephrine (mcg/h)<br>o intrathoracic blood volume index (ITBVI) and extravascular lung water index (EVLWI), (PICCO)<br>▪ Time on ventilator *(measured from intubation in hours; the need of reintubation will be documented)*)<br>▪ Requirements of *postoperative* ventilatory support (PCV, CPAP)<br>▪ Nursing Delirium Screening scale (NuDesc) – *measurement three times daily on ICU**<br>▪ gastrointestinal parameters - (*measurement daily from post-op day +1 until fulfilment or until end of study, whatever occurs first)*<br>o time until bowel movements (on auscultation)<br>o time until full oral nutrition<br>o time to first flatus<br>o time to first defecation<br>o intraabdominal pressure *measured baseline (after induction of anaesthesia), at the end of surgery (still in operating theatre before extubation), 6 hours after surgery, 1st post-operative day in the morning*<br>▪ SOFA, SAPS II, APACHE II Scores – *surveyed once daily on ICU**<br>▪ length of stay in ICU/IMCU (fulfillment of ICU/IMCU release criteria according to the Aldrete Score, Aldrete 1995) *surveyed on admission to ICU, 1st postoperative day in the morning, at time of discharge from ICU*<br>▪ time until criteria for “fit for discharge from hospital“ are fulfilled (hospitalization release criteria according to Marshall et al., 1999, Marshall and Chung, 1997) *surveyed once daily on ICU respectively surgical ward (if fit-for-discharge criteria are not fulfilled within the 5th postoperative day it will be controlled until fulfillment or until end of study, whatever occurs first)*<br>▪ Complication rate - surgical complications such as re-do surgery (e. g. anastomotic insufficiency or bowel leakage), disturbed wound-healing or medical complications which delay time to fully oral nutrition – *documentation at the study end*<br>▪ combined outcome parameter of prolonged length of stay (based on mean length of stay according to the German Diagnosis Related Group System D-DRG) or death (determination will be when DRG-data are available, i. e. after study end)<br>▪ Health Related Quality of Life (HRQoL) according to the EQ-5D questionaire – *measurement baseline and 3 months after surgery*

	* if not otherwise mentioned measurements of secondary variables on ICU will be performed until fit-for-discharge criteria are fulfilled or until postoperative day 5 (including), whatever occurs first.<br><br>Other<br>▪ Demographic data<br>▪ Surgery related data (duration of surgery and anaesthesia, precise classification of surgical procedure)<br>▪ Concomitant medication/therapy *(pre-operative and pre-surgery, during surgery, on ICU**)
Patient Inclusion / Exclusion Criteria	Inclusion criteria:<br>▪ Male or female patients ≥ 18 years of age and ≤ 80 years of age. Women of child bearing potential must test negative on standard pregnancy test (urine).<br>▪ Patients scheduled to undergo planned elective surgery of the pancreatic head (e.g. PPPD)<br>▪ Patients who are willing to give of voluntary consent to participate in the study, following a full explanation of the nature and purpose of the study, by signing the informed consent form approved by the Institutional Ethics Committee (IEC) prior to all evaluations.<br>Exclusion criteria:<br>▪ Patients of ASA-class > III<br>▪ Heart failure defined as NYHA class>2<br>▪ Aneurysm of the ascending and thoracic aorta<br>▪ Patients with Zenker's diverticle<br>▪ Local oesophageal disease (oesophageal stricture, oesophageal varices, previous oesophageal surgery in past 6 months before study inclusion, pharyngeal pouch)<br>▪ Patients receiving haemodialysis<br>▪ Patients with known bleeding diatheses<br>▪ Any bleeding disorder known from patient's history<br>▪ Patients with a haematocrit ≤ 25% despite pre-op transfusion<br>▪ Renal insufficiency (serum creatinine > 130 µmol/l or >1.5 mg/dl) or oliguria or anuria<br>▪ Impaired hepatic function as expressed by a decreased Quick-value of < 60% or liver cirrhosis Child-Pugh C<br>▪ Additional contra-indications for either Tetraspan 6%, Tetraspan 10% and Sterofundin Iso are<br>o hyperhydration state incl. lung oedema, generalized oedema, hypervolaemia<br>o intracranial haemorrhage

<table>
<tr><td></td><td>
o hyperkalaemia, severe hypernatraemia or severe hyperchloraemia, hypercalcaemia
o known hypersensitivity to HES or any of the excipients
▪ Pregnancy or lactation period
▪ Simultaneous participation in another interventional clinical trial (drugs or medical devices)
▪ Emergencies
▪ By judicial or enforcable order detained patients
</td></tr>
<tr><td>Investigational Test Product</td><td>
The Investigational Test Product (Tetraspan 10%) contains 100 g/l of hydroxyethyl starch (HES) with a mean molecular weight of 130 kDalton, a molar substitution of 0.42 and a substitution ratio of 6:1 which is dissolved in plasma adapted Ringer’s solution (balanced solution).

Composition:
1000 ml solution contain
hydroxyethyl starch (HES 130/0.42) 100 g
sodium chloride 6.25 g
potassium chloride 0.30 g
calcium chloride dihydrate 0.37 g
magnesium chloride hexahydrate 0.20 g
sodium acetate trihydrate 3.27 g
malic acid 0.67 g

electrolyte concentration
sodium 140.0 mmol/l
potassium 4.0 mmol/l
calcium 2.5 mmol/l
magnesium 1.0 mmol/l
Chloride 118.0 mmol/l
acetate 24.0 mmol/l
malic acid 5.0 mmol/

pH: 5.6 – 6.4
theoretical osmolarity: 297 mOsmol/l
titration acidity: 2.0 mmol/l
</td></tr>
<tr><td>Investigational Reference Product</td><td>
The Investigational Reference Product (Tetraspan 6%) differs from the Investigational Test Product (Tetraspan 10%) solely with regard to the concentration of HES. It contains 60 g/l of hydroxyethyl starch (HES) with a mean molecular weight of 130 kDalton, a molar substitution of 0.42 and a substitution ratio of 6:1 which is dissolved in plasma adapted Ringer’s solution (balanced solution).

Composition:
1000 ml solution contain
hydroxyethyl starch (HES 130/0.42) 60 g
sodium chloride 6.25 g
potassium chloride 0.30 g
calcium chloride dihydrate 0.37 g
magnesium chloride hexahydrate 0.20 g
sodium acetate trihydrate 3.27 g
malic acid 0.67 g

electrolyte concentration
sodium 140.0 mmol/l
potassium 4.0 mmol/l
calcium 2.5 mmol/l
magnesium 1.0 mmol/l
</td></tr>
</table>

<table>
<tr><td></td><td>
Chloride 118.0 mmol/l
acetate 24.0 mmol/l
malic acid 5.0 mmol/

pH: 5.6 – 6.4
theoretical osmolarity: 296 mOsmol/l
titration acidity: 2.0 mmol/l
</td></tr>
<tr><td>Descriptive Control Treatment Group</td><td>
Furthermore, comparison will be made to the infusion of solely an electrolyte solution (crystalloid): Sterofundin ISO which is a plasma adapted Ringer's solution (balanced electrolyte solution)

Composition:
1000 ml solution contain
sodium chloride 6.80 g
potassium chloride 0.30 g
calcium chloride dihydrate 0.37 g
magnesium chloride hexahydrate 0.20 g
sodium acetate trihydrate 3.27 g
malic acid 0.67 g

electrolyte concentration
sodium 140.0 mmol/l 145 mmol/l*
potassium 4.0 mmol/l .
calcium 2.5 mmol/l .
magnesium 1.0 mmol/l .
Chloride 127.0 mmol/l .
acetate 24.0 mmol/l .
malic acid 5.0 mmol/ .

pH: 5.1 – 5.9
theoretical osmolarity: 309 mOsmol/l
titration acidity: ~ 5 mmol/l
</td></tr>
<tr><td>Investigational Products Administration</td><td>
Method of Administration

The administration of the Investigational Products is performed intravenously.

Dosage (intraoperatively)

Administration of Investigational Test and Reference Products will be performed as an "add on" volume administration to a continuous basal crystalloid infusion. The continuous basal infusion of the crystalloid will be 4 ml/kg bodyweight/h. Administration of crystalloid basal infusion will start at induction of anaesthesia and will be continued throughout surgery. A balanced crystalloid solution will be used for basal infusion (Sterofundin ISO).

The "add on" fluid/volume administration of either HES 10%, HES 6% or balanced crystalloid in a blinded manner will individually be controlled by measuring stroke volume with oesophageal Doppler to achieve a target controlled cardiac preload optimum during surgery. Depending on change of stroke volume bolus infusions of 250 ml/5 min will be administered. A detailed algorithm (flow diagram) will provide basis for amount of volume replacement.
</td></tr>
</table>

	In case intra-operative volume requirements exceed the amount of bottles of Investigational Product supplied by the pharmacy in a blinded manner then open-label treatment with a balanced crystalloid solution (Sterofundin ISO) will be performed during surgery (also using oesophageal Doppler measurements for deciding on the administration of the fluids).<br><br>All Investigational Products will be administered in a blinded manner. Blinding will be performed by the pharmacy who will compile the appropriate amount of bottles and label with sequential numbers.<br><br>Two independent anaesthesists will be involved to maintain blindness: one for maintenance of anaesthesia, one for Doppler measurements and volume and haemodynamic therapy.<br><br>Treatment Duration<br><br>Treatment with Investigational Product starts after reliable basal oesophageal Doppler measurements are obtained and before skin incision.<br><br>After final positioning of the patient in the operating theatre the oesophageal probe will be placed and basal measurements will be recorded after correct placement of probe and reliable measures are obtained. Thereafter, before skin incision, the first bolus of Investigational Product of 250 ml/5 min will be given and the algorithm of the individualized volume replacement therapy is initiated. Oesophageal Doppler guided volume replacement will be performed throughout surgery.<br><br>Treatment with Investigational Product ends with surgery when the oesophageal probe is withdrawn. In case all bottles of Investigational Product as compiled by the pharmacist are used up before end of surgery then open-label treatment with a balanced crystalloid (Sterofundin ISO) will be performed from then on during surgery.<br><br>After surgery, on ICU, volume replacement will be performed at the discretion of the attending physician. In case colloidal volume replacement is required on ICU on the day of surgery no HES-product but other colloidal volume replacement (e. g. gelatine) will be used. All volume/fluid replacement therapy will be recorded (separately for colloids and crystalloids).<br><br>Trigger for transfusion of erythrocyte concentrate (EC) is an haematocrit of < 25% (or if necessary). Infusion of fresh frozen plasma (FFP) will aim at a proportion of EC:FFP=2:1 and 1:1 after 4 infusions of EC respectively. Trigger for infusion of patelet concentrate (PC) is a thrombocyte count of < 50.000/µl.
Visit Schedule	day -1(range -5)/ pre-operatively:<br>▪ Verification of inclusion and exclusion criteria, including Informed consent<br>▪ Randomization<br>▪ History, demographics, body weight, body temperature<br>▪ HRQoL (EQ-5D questionnaire)<br>▪ Pre-operative medication / therapy as medically indicated and standardized (including pre-operative

	fasting time, type of bowel preparation)<br>o Pre-op transfusion will be administered according to standard procedure (hct. between 25 and 30%)<br><br>day 0/ prior anaesthesia:<br>▪ start basal infusion of crystalloid fluid replacement: 4 ml/kg/h<br>▪ insert thoracic epidural catheter<br>▪ induction anaesthesia<br><br>day 0/ after induction of anaesthesia/prior surgery :<br>▪ set arterial line and central venous catheter<br>▪ set of urethral catheter and measurement of intraabdominal pressure<br>▪ final positioning of patient in operating theatre<br>Note: Loading of epidural analgesia starts at the end of surgery when all study related intraoperative measurements have been performed<br>▪ placement of oesophageal probe, calibration of PiCCO<br>▪ baseline recording of haemodynamics (SAP, DAP, MAP, HR, CVP; oesophageal Doppler and PiCCO: CI, SV, SVR; PiCCO: ITBVI, EVLWI)<br>▪ Blood sampling and urine sample for laboratory evaluation*<br>▪ Concomitant medication and therapy (including concomitant medication prior induction of anaesthesia)<br><br>*Baseline laboratory<br>o arterial blood gas analysis (pCO2, pO2, HCO3, SaO2) including acid base status (pH, BE, lactate, bicarbonate) and electrolytes (Na, K, Ca, Cl). Blood sampling for BGA should be performed as close as possible to the time, at which the corresponding hemodynamic measurement is done.<br>o renal function parameters (*urine*: α1-MG, β-NaG, creatinine, *serum*: creatinine, BUN, HbA1c)<br>o haemostasis and coagulation parameter (PT, aPTT, Fibrinogen, vWF-Ag, vWF-RiCo, F VIII:c; CT, CFT, MCF using ExTEM und FibTEM)<br>o Inflammamatory parameters (IL6 (facultative), IL 10 (facultative), PCT, CRP, white blood cell count (WBC))<br>o cardiac function parameters (Troponin or high-sensitivity Troponin, NT-Pro-BNP)<br><br>day 0/ after baseline recordings:<br>▪ first bolus (250 ml/5 min) of Investigational Products<br>▪ start of volume replacement algorithm according to oesophageal Doppler measurements<br>▪ thereafter: start of surgery/skin incision<br><br>day 0/ during surgery (after start of skin incision):<br>▪ Haemodynamics<br>o *measurements following dosing algorithm (i.e. in 15 min intervals when no volume optimisation, or 15 minutes after new optimised stroke volume):*

	SAP, DAP, MAP, HR, CVP; PiCCO and oesophageal Doppler: CI, SV, SVR<br>o *every 60 min after start of surgery*: ITBVI, EVLWI (PiCCO)<br>▪ Infusion of the investigational Products according to algorithm<br>▪ Hourly blood sampling for intraoperative laboratory evaluation*<br>▪ Diuresis/urinary output (cumulative every 60 min)<br>▪ Concomitant medication and therapy (including inotropic drugs, sodium bicarbonate, infusion of blood products (RBC, FFP, PC) according to the triggers<br>▪ Adverse events and adverse reactions<br>*laboratory measurements in blood samples:<br>o 1 hour after start of surgery: haemostasis/ coagulation (ROTEM only), blood gas analysis/arterial blood sample (pCO2, pO2, HCO3, SaO2) including acid base status (pH, BE, lactate, bicarbonate) and electrolytes (Na, K, Ca, Cl)<br>o at 2 hours after start of surgery and then every 60 min intraoperatively: blood gas analysis/arterial blood sample (pCO2, pO2, HCO3, SaO2) including acid base status (pH, BE, lactate, bicarbonate) and electrolytes (Na, K, Ca, Cl).<br>o blood sampling for BGA should be performed as close as possible to the time, at which the corresponding hemodynamic measurement is done. i.e. ITBVI and EVLWI.<br>day 0/ at the end of surgery:<br>▪ Haemodynamics (SAP, DAP, MAP, HR, CVP; PiCCO and oesophageal Doppler: CI, SV, SVR; PiCCO: ITBVI, EVLWI)<br>▪ intraabdominal pressure (still in operating theatre directly before extubation)<br>▪ Concomitant medication and therapy, blood products<br>▪ Blood sampling for laboratory evaluation*<br>▪ Investigational Products<br>▪ Diuresis/urinary output<br>▪ Adverse events and adverse reactions<br>* laboratory measurements in blood sample:<br>o arterial blood gas analysis (pCO2, pO2, HCO3, SaO2) including acid base status (pH, BE, lactate, bicarbonate) and electrolytes (Na, K, Ca, Cl). Blood sampling for BGA should be performed as close as possible to the time, at which the corresponding hemodynamic measurement is done.<br>o haemostasis/coagulation parameters (PT, aPTT, Fibrinogen, FVIII:c, vWF-Ag, vWF-RiCo; CT, CFT, MCF using ExTEM und FibTEM)<br>o cardiac function (Troponin or high-sensitivity Troponin, NT-Pro-BNP)<br>o inflammatory reaction parameters (facultative: IL-6, IL-10)

	day 0/ intensive care unit (ICU) –<br>▪ Aldrete Score (*on admission)*<br>▪ Haemodynamics (SAP, DAP, MAP, HR, CVP) on admission and 6 hourly.; PiCCO: (CI, SV, SVR, ITBVI, EVLWI*) on admission and after 6 hours*)<br>▪ Blood gas analysis (including acid base status and electrolytes) on *admission and 6 hours thereafter.* Blood sampling for BGA should be performed as close as possible to the time, at which the corresponding hemodynamic measurement is done.<br>▪ abdominal pressure *(6 hours after admission)*<br>▪ Ventilatory support as requested to maintain normocapnia and oxygen saturation (PCV, CPAP)<br>▪ Concomitant medication, infusion therapy (separate for colloids (i. e. gelatine), crystalloids, blood products)<br>▪ Diuresis (until next morning about 6 o'clock)<br>▪ Adverse events and adverse reactions<br>▪ check of discharge criteria<br><br>day +1 (1st post-operative day)/ On ICU: Postoperative care will follow a standardised regimen.<br>▪ Haemodynamics: SAP, DAP, MAP, HR, CVP (*every 6 hours*); PiCCO: CI, SV, SVR, ITBVI, EVLWI (every 12 hours)<br>▪ abdominal pressure (in the morning)<br>▪ Ventilatory support (PCV, CPAP) as requested to maintain normocapnia and oxygen saturation<br>▪ Blood sampling for laboratory evaluation*, urine sample for laboratory evaluation**<br>▪ Aldrete Score<br>▪ SIRS-criteria<br>▪ NuDesc (*3x daily)*<br>▪ Severity of injury scores (APACHE II, SAPS II, SOFA)<br>▪ Assessment of "fit for discharge" criteria (once on ICU-preferably in the morning)<br>▪ Concomitant medication including parenteral nutrition, infusion therapy (separate for colloids i. e. gelatine and crystalloid, blood products)<br>▪ Diuresis (sampling period from morning until next morning about 6 o'clock)<br>▪ PONV<br>▪ gastrointestinal parameters<br>o bowel movements on auscultation<br>o time until full oral nutrition<br>o time until first flatus<br>o time until first defecation<br>▪ Surgical complications (anastomotic insufficiency, intestinal leakage, wound healing)<br>▪ body weight (if possible)<br>▪ Adverse events and adverse reactions<br>▪ check of discharge criteria<br><br>* laboratory measurements in blood samples 1st post-operative day:<br>o in the morning sample: Troponin or high-sensitivity Troponin, NT-Pro-BNP, IL6

	(facultative), IL10 (facultative), PCT, CRP, WBC, creatinine, BUN, PT, aPTT, vWF-Ag, vWF-CoRi, ROTEM, platelet count<br>o every 6 hours: blood gas analysis/acid base status/electrolytes. Blood sampling for BGA should be performed as close as possible to the time, at which the corresponding hemodynamic (SAP, DAP, MAP, HR and CVP) measurements are done.<br><br>**laboratory measurements in urine sample: α1-MG, creatinine, ß-NAG<br><br>day +2 – day +n, until fulfillment of "fit for discharge"-criteria (Marshall and Chung 1997, 1999) however not longer than until day +5 (including day +5) (blood sampling also on (postoperative) day +3 and +7)<br>▪ Haemodynamics (SAP, DAP, MAP, HR, CVP(every 6 hours); PiCCO: CI, SV, SVR; ITBVI, EVLWI (every 12 hours))<br>▪ Ventilatory support (PCV, CPAP) as requested to maintain normocapnia and oxygen saturation<br>▪ Blood sampling/urine sampling for laboratory evaluation (daily)*<br>▪ SIRS-criteria<br>▪ NuDesc (3x daily)<br>▪ Severity of injury scores (APACHE II, SAPS II, SOFA) (once daily)<br>▪ Assessment of "fit for discharge" criteria (once on ICU- preferably in the morning)<br>▪ Concomitant medication, infusion therapy (separate for colloid (gelatine) and crystalloid, blood products)<br>▪ Diuresis (sampling period from morning until next morning about 6 o'clock)<br>▪ PONV<br>▪ Surgical complications (anastomotic insufficiency, intestinal leakage, wound healing)<br>▪ Gastrointestinal parameters (bowel movement on auscultation, start of enteral nutrition, time to first defaecation)<br>▪ Mobilisation<br>▪ "Fit for discharge"-criteria (Marshall and Chung, 1997, 1999) : fit for discharge criteria will be controlled until fulfilled or until study end, whatever occurs first<br>▪ Aldrete Score (assessment at time when discharge from ICU)<br>▪ Adverse events and adverse reactions (continuously until postoperative day 5, serious adverse events that are not yet resolved on POD 5 will be followed up until study end)<br><br>*laboratory measurements on ICU, respectively on (postoperative) days +3 and +7<br>o once daily on ICU (max. for 5 days): renal function parameters (blood: creatinine, BUN), platelet count, WBC, blood gas analysis (pH, HCO3, sodium, potassium, Hct) for scores; urine:

	α1-MG, ß-NAG, creatinine<br>o postoperative days 3 and 7: creatinine in serum<br><br>day +2 – day +15 maximal (i. e. as long as patient receives parenteral/enteral nutrition) on respective care unit (control on subsequent day for predecessor day)<br>▪ control of nutrition/diet: Is parenteral nutrition administered – Has the patient taken nutrients orally at least for three times that day.<br>3 months after surgery<br>▪ Health Related Quality of Life (HRQoL)
Duration of Study per Patient	Start of study: with randomisation<br>Treatment period (infusion of Investigational Products):<br>During surgery (guided by measurement of oesophageal Doppler)<br>End of study: Postoperative Day 15 or discharge from hospital whatever occurs first<br>Last evaluation of certain parameters will be 3 months post-surgery respectively when DRG data are available
Study Schedule	Planned start: June 2010<br><br>Planned recruitment time: 2 years<br><br>Planned last patient out: May 2012
Statistical Methods	For the multiple primary endpoint the principle of ordered hypothesis will be used for testing. If the first primary hypothesis will result in a statistically significant difference the second primary variable will be analyzed using the same procedure (with an unchanged error of the 1st kind α = 5%) as described for the first primary variable.<br><br>The two primary endpoints will be tested for difference between the two HES-groups using a non-parametric statistical test (Mann-Whitney U-Test). This test is chosen with regard to small sample sizes and possible deviation from normal distribution.<br><br>All other parameters will be analysed exploratively and descriptively. This analysis will also serve for testing of homogeneity of treatment groups at baseline.<br><br>All tests will be performed two-sided with an error of 1st kind α = 5%.
Randomization / Blinding	The patients will be randomised to either treatment in a 1:1:1 ratio. Randomisation will be performed in permuted blocks.<br><br>The population will be stratified with regard to<br>▪ centre<br>▪ ASA-class (2 strata): ASA≤2 and ASA=3

	Baseline fluid administration with crystalloid will be performed open-label while add-on volume replacement with either balanced HES 10%, 6% or crystalloid will be performed double-blind.
Drug Safety Monitoring Board (DSMB)	not applicable

4 INTRODUCTION

The aim of volume replacement is to compensate a reduction in the intravascular volume e. g. during surgery and to counteract hypovolaemia in order to maintain haemodynamics and vital functions. Boldt (Boldt, 2005) concluded that volume replacement should not only tend to optimize macrocirculation and oxygen delivery but also to improve organ perfusion, microcirculation and inflammatory response.

Hydroxyethyl starch (HES) is the most intensively studied artificial colloidal plasma volume substitute (Boldt & Suttner, 2005). It consists of hydroxyethylated polymers of glucose, derived from amylopectin. The characteristics of HES can mainly be defined by the molecular weight and the molar substitution ratio, i.e. the ratio of replacement of glucose group by hydroxyethyl group during production. Various HES preparations exist showing different physico-chemical properties ((Jungheinrich & Neff, 2005),(Boldt & Suttner, 2005)). The Investigational Products Tetraspan® contain HES with an average molecular weight of 130,000 Daltons, a molar substitution of 0.42 and a substitution ratio of 6:1 is solved in a balanced electrolyte solution. The cation pattern in the crystalloid component Tetraspan® is adapted to physiological plasma electrolyte concentrations. The anion pattern is a combination of chloride, acetate and malate. The purpose is to minimize the risk of hyperchloraemia and acidosis. The additions of acetate and malate instead of lactate anions are intended to reduce the risk of lactacidosis. In conclusion, Tetraspan® combines benefits of the modern 3. generation HES 130/0.42 with proven advantages of a balanced plasma adapted electrolyte solution.

While Tetraspan® 6% (containing 6% HES) is iso-oncotic, i.e. the increase in the intravascular plasma volume is equivalent (i. e. 100%) to the infused volume, the Investigational Test Product Tetraspan® 10% (containing 10% HES) is hyper-oncotic, i. e. the volume effect is 145%. Thus, a considerable intravasal effect could be achieved without overloading the interstitial compartment with fluid and consequently reducing oedema tendency with it's known pathophysiological consequences ((Lobo *et al*, 2006), (Brandstrup, 2006)).

All products used in the present phase IV trial are registered in Germany as well as in various countries in Europe. The clinical use of the Investigational Products in the present study is within the approved Summary of Product Characteristics (SmPC).

5 STUDY RATIONALE

In elective pancreatic surgery intra- and postoperative volume replacement requirements are considerably high. With the administration of a hyper-oncotic colloid maintenance of haemodynamics and vital functions could be achieved more easily than with an iso-oncotic colloid or sole crystalloid therapy avoiding an fluid overload of the interstitial compartment which could lead to oedema tendency with it's known pathophysiological consequences.

Elective pancreatic surgery is characterized by a highly standardized surgical, anaesthetic and postoperative care (SOP Dept. of Anaesthesiology and Intensive Care Medicine) and is well suited to compare the volume requirements following hyper-oncotic or iso-oncotic volume replacement therapy in comparison to crystalloid volume replacement alone. Individual intraoperative volume replacement therapy during surgery can be improved by target controlled replacement using oesophageal Doppler measurement as has been shown e. g. for abdominal surgery (Noblett et al. 2006, Wakeling et al. 2005).

6 RISK-BENEFIT-ASSESSMENT

All Investigational Products are registered in Germany as well as in various European countries. The proposed use of the Investigational Products in the present study is in line with the registered SmPCs.

Goal-directed perioperative fluid therapy by determination of stroke volume has been found to reduce the risk of hypovolaemia or hypervolaemia and to reduce postoperative complications as well as length of stay in hospitals. This favours the use of individualized goal directed fluid and volume replacement therapy with oesophageal Doppler measurements (Review of the Agency for Healthcare Research and Quality Technology, 2007) instead of determining blood pressure alone as the ground for deciding volume administration.

Many of the proposed measures and parameters (laboratory, clinical) are performed/obtained routinely and thus, do not represent major additional burden to the patient.

In conclusion, the risk due to methods and measurements in the present study is considered to be low and a positive risk-benefit is assessed.

7 STUDY OBJECTIVES

7.1 Primary Objective

It is the primary aim of the planned study to investigate on the efficacy of target controlled fluid therapy with a hyper-oncotic compared to an iso-oncotic balanced HES 130/0.42 solution in adult patients undergoing elective pancreatic surgery.
.

7.1.1 Primary Variable

A multiple primary endpoint is defined which is "the intra-operative amount (in mLs) of colloidal volume replacement therapy during target directed volume replacement therapy" as the first primary endpoint and "days until fully on oral (solid) diet" as the second primary endpoint (full on oral diet is defined by the absence of parenteral and enteral nutrition and the patient is able to take oral nutrition for at least three times that day).

7.2 Secondary Objectives

Secondary objectives are the investigation of safety and efficacy parameters like blood coagulation, perioperative blood loss, anastomotic or bowel leakage, renal function, haemodynamics, postoperative inflammation, intraabdominal pressure, adverse events and amount of given blood products (RBC, FFP, PC). Furthermore, descriptive comparison of the hyper- and iso-oncotic volume replacement therapy with HES to volume replacement therapy with a crystalloid alone will be made.

8 STUDY DESIGN

This is a prospective, randomized, controlled, double-blind, multi-centre phase IV study performed in three parallel groups.

Comparison will be made of a hyper-oncotic (10%) balanced HES 130/0.42 solution with an iso-oncotic (6%) balanced HES 130/0.42 solution on the basis of a multiple endpoint. The principle of ordered hypothesis will be used [see section 16 statistics]

for testing the two primary endpoints: “the intra-operative amount of colloidal volume replacement therapy during target directed volume replacement therapy” (first), subsequently “days until fully on oral solid diet” (second).

A third treatment arm (as a descriptive control) will comprise the administration of solely a balanced crystalloid fluid replacement. This group is included in the study as in various hospitals and countries respectively volume replacement solely with electrolytes is still a standard in surgery including pancreatic surgery. As crystalloid solutions, however, display another volume effect* than colloidal solutions (*thus leading to to a distinct higher requirement of volume which could be easily recognised even when blinded) this treatment group will only serve as a descriptive control.

The study will be started with an internal pilot phase which serves for recalculation of sample size for the first primary variable respectively for ensuring sufficient power for the second primary variable. Interim analysis will also serve for deciding of premature termination of the study for futility. This interim analysis will be performed by estimating the pooled variances for both primary endpoints without unblinding [see section 16 statistics].

In the first phase of the study (internal pilot phase) the number or patients is 20 (including drop-outs) for each treatment arm.

Beside this two-phase design with a number of 60 patients in the pilot phase, a rough estimation of the sample size (for the 1st primary variable) to expect resulted in:

69 patients per group, supposing an effect size (difference of means)/(common standard deviation) = ½ = 0.5 , error of the 1st kind alpha = 5% (two-sided), Power = 80% and the nonparametric Mann-Whitney U-Test (Calculations with nQuery Advisor, version 6.0). Taking a drop-out rate of 10% into consideration, a sample size of 76 patients per group will be needed.

This sample size also should be sufficient for the 2nd primary variable (for details see section 16)

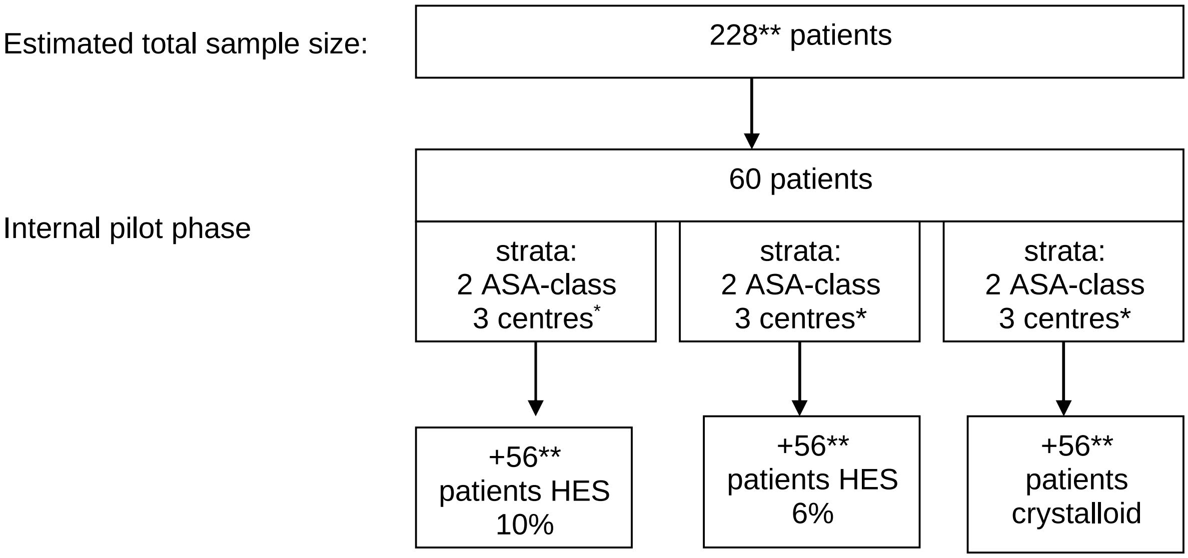

The study will be performed in three study centres in Berlin and Bonn, Germany.

** may change according to calculation of pooled variance after internal pilot phase.

9 SELECTION AND WITHDRAWAL OF PATIENTS

9.1 Informed Consent

An informed consent, written in accordance with the origins of the Declaration of Helsinki (Appendix 1) and the applicable laws of the country has to be obtained from all patients.

The patient will sign the Informed Consent Form *(*Appendix 2*)* before s/he enters the study, i.e. before screening bloods, screening assessments or any other study-related activity. The Investigator will explain the nature, purpose and risks of the study including data confidentiality and protection aspects and provide the patient with a copy of the patient information *(*Appendix 2*)*. The patient will be given sufficient time (preferably one day in advance) to consider the study's implications before deciding whether to participate.

Should there be any amendments to the Final Protocol, such that would directly affect the patient's participation in the study e.g. a change in any procedure, the Informed Consent Form must be amended to incorporate this modification and the patient's informed *re*-consent must be obtained.

9.2 Patient Inclusion Criteria

The following inclusion criteria apply:

- Male or female patients ≥ 18 years of age and ≤ 80 years of age. Women of child bearing potential must test negative on standard pregnancy test (urine).
- Patients scheduled for undergoing planned elective surgery of the pancreatic head
- Patients who are willing to give of voluntary consent to participate in the study, following a full explanation of the nature and purpose of the study, by signing the informed consent form approved by the Institutional Ethics Committee (IEC) prior to all evaluations.

The investigator will maintain a screening log (appendix 3) on all patients considered for inclusion into the study in the Investigator Site File

9.3 Patient Exclusion Criteria

Patients will not be considered for participation in the study if any of the following criteria listed below apply:

- Patients of ASA-class > III
- Heart failure defined as NYHA class>2
- Aneurysm of the ascending and thoracic aorta
- Patients with Zenker's diverticle
- Local oesophageal disease (oesophageal stricture, oesophageal varices, previous oesophageal surgery in past 6 months before study inclusion, pharyngeal pouch)
- Patients receiving haemodialysis
- Patients with known bleeding diatheses
- Any bleeding disorder known from patient's history
- Patients with a haematocrit ≤ 25% despite pre-op transfusion

- Renal insufficiency (serum creatinine > 130 µmol/l or >1,5 mg/dl) or oliguria or anuria
- Impaired hepatic function described by decreased Quick-value < 60% or liver cirrhosis Child-Pugh C
- Additional contra-indications for either Tetraspan 6%, Tetraspan 10% and Sterofundin Iso are
  - hyperhydration state incl. lung oedema, generalized oedema, hypervolaemia
  - intracranial haemorrhage
  - hyperkalaemia, severe hypernatraemia or severe hyperchloraemia, hypercalcaemia
  - known hypersensitivity to HES or any of the excipients
- Pregnancy or lactation period
- Simultaneous participation in another interventional clinical trial (drugs or medical devices studies).
- Emergencies
- By judicial or enforcable order detained patients.

9.4 Stopping and Discontinuation Criteria

When the study in an individual patient is terminated, the nature of termination must be documented (scheduled end or premature termination/discontinuation). In the event of premature termination/discontinuation of the study, justification has to be given and it is to be recorded who took the decision to discontinue.

If the study as a whole is prematurely terminated or suspended, the concerned IEC and the regulatory authorities will be informed promptly and provided with the reasons for the termination or suspension by the sponsor.

9.4.1 Discontinuation Criteria Related to the Study

Criteria for discontinuation / termination of the entire clinical study include (not exclusively)

- Unexpected high frequency of serious adverse reactions during the study (frequency as outlined in SmPC will serve as a basis).
- Occurrence of a suspected unexpected serious adverse reaction which does not justify a continuation of the study
- Patients cannot be recruited in sufficient numbers. Not sufficient is defined as recruitment of less than 4 patients per month on average in all participating centres.

If after pilotphase (after 60 patients) the calculation of the pooled variance reveals that the estimated total number of 228 patients will be exceeded the study will not be carried on but stopped for futility after the pilot phase (see section 16).

9.4.2 Discontinuation Criteria Related to the Study Site

Discontinuation of the clinical study in an individual study site may occur because of various reasons including

- Failure of Investigator to comply with the ICH-GCP and/or applicable regulatory requirements.

- Submission of knowingly false or incomplete information from the site to B|Braun Melsungen AG, Study Monitor, or the authorities.
- Repeated non-adherence to protocol requirements including insufficient data quality (missing data in case report forms (CRFs) occurring repeatedly)
- Failure of the Investigator at a site to enrol patients into the study at an acceptable rate, i.e. less than 9 patients per 3 months.
- Personnel change without appropriate information to the sponsor.

9.4.3 Discontinuation Criteria related to the Patient

The patients will be advised in the Informed Consent Forms that they have the right to withdraw from the study at any time without prejudice, and may be withdrawn at the Investigator's / Sponsor's discretion at any time, when this is considered to be in the interest of the patient.

Withdrawal of individual patients from treatment or from the study respectively could be caused by the following reasons (not exclusively):

Withdrawn by the Investigator due to

- Adverse Event, Serious Adverse Event (including pregnancy), e. g. hypersensitivity to HES, clinical significant abnormal laboratory value(s) such as hyperkalaemia (> 5,8 mmol/l), severe hypernatraemia (> 155 mmol/l) or severe hyperchloraemia (> 125 mmol/l), hypercalcaemia (> 2,8 mmol/l)
- The haemodynamic monitoring for guiding the application of the study drugs cannot be established.
- Protocol deviation or failure to comply with the protocol (e.g. dosing regimen, administration of an excluded medication, surgery performed as explorative laparotomy/premature termination of surgery)
- Subsequent occurrence of an exclusion criterion

which leads to inacceptance of further remaining either on treatment or in the study as considered by the investigator.

The patient requested withdrawal due to:

- An Adverse Event for which the Investigator did not consider removal from the treatment/study necessary
- Perceived insufficient therapeutic effect
- Perceived sufficient therapeutic effect
- Withdrawal of consent

In case a patient does not reappear to any scheduled visit (i. e. is lost to follow-up) reasonable effort should be made to contact this patient in order to complete assessments and/or to evaluate reason for non-appearance (possibly implicit withdrawal of consent).

In the event that a patient withdraws consent or is withdrawn from the *study*, the study termination page in the CRF should be completed indicating that the study was prematurely terminated. The Investigator should record the date of the withdrawal, the person who initiated withdrawal and the reason for withdrawal.

9.5 Randomisation, Blinding and Unblinding

Patients will be randomized to either treatment in a 1:1:1 ratio.

The list of treatment assignments considering the stratification for investigational centre and ASA-class (2 strata: ASA≤2 and ASA=3) will be generated by an independant study statistician comprised of consecutive blocks with the order of assignments chosen at random (i.e.: random permuted block of size 3, 6 or 9). The list will be prepared prior to the initiation of the study and will remain with the statistician. Based on this list the statistician will issue sets of random envelopes for each participating hospital, i. e. for the hospital's pharmacies for allocation the patient to the respective treatment and for the Principal Investigators for emergency unblinding (see below). The sponsor will receive a complete set of random envelopes also for emergency situations.

Patients who are eligible for inclusion into the study shall receive an increasing sequential patient number (enrolment number) in accordance with the order of their inclusion in the study. A random number will be issued which includes centre number and strata (A for ASA ≤ II, B for ASA = III) and and increasing consecutive number (within the ASA-class stratum). Enrolment of patients will be recorded in the enrolment log (appendix 4: a for stratum A and b for stratum B) A randomization sheet (appendix 5) will be issued with patient's data essential for compilation of the appropriate Investigational Product (Patient ID number, name and birthday – either hand written or use of hospital ID-label –, random number, ASA-class, body weight) and forwarded to the pharmacy to compile the appropriate amount of the respective Investigational Product.

Blinding will be performed by the hospital's pharmacy. In order to maintain blinding the following procedure has to be followed: The maximal possible daily dose for the 6% HES solution is determining the amount of bottles to be compiled for all treatment groups. In the group with HES 10% a balanced crystalloid fluid will be administered (in blinded manner) when the maximum daily dose of HES is reached. Thus, it will be ensured that in all treatment groups are the same amount of bottles (depending on bodyweight).

Example for a 70 kg patient: The maximum daily dose for HES 6% is 50ml x 70kg/d = 3500ml/d. Thus, 7 bottles with 500ml have to be compiled and will be labelled with sequential number 1 - 7. If according to the stratified randomization list the 70 kg patient has to receive treatment with HES 10% (i. e. maximum daily dose is 30ml x 70kg/d = 2100ml) the pharmacist will compile 4 bottles of HES 10% which are labelled with sequential number 1 – 4 and further 3 bottles of crystalloid labelled as bottles 5 – 7. (Note: as bottles contain always 500ml and maximum daily dose of HES is not to be exceeded the amount has to be appropriately rounded down). If according to the stratified randomization list the patient has to be treated with the crystalloid replacement therapy 7 units (numbered 1 – 7) of the electrolyte product will be used.

Bottles always have to be given to the patient in sequential order. This is essential in case not all bottles are required for replacement therapy during surgery and has impact for the group with HES 10%.

For emergency unblinding the statistician will provide sealed envelopes with the patient random number printed to the Principal Investigator (each centre). The envelopes will be sent to the study centre prior start of the study. When breaking the seal of an envelope the respective treatment for the individual patient is stated. The code may only be broken when this is relevant for the safety of the trial subject. Any premature code break (e.g. unblinding due to a Serious Adverse Event, accidental unblinding) should promptly be documented and reported to the Sponsor. Emergency

unblinding may be discussed with the Sponsor prior breaking the seal if possible, i. e. if this is not affecting the patient's safety. Upon study completion all emergency envelopes will be retrieved by the study monitor.

After closure of the database and determination of the analysis populations (in a blinded data review meeting if appropriate) the study will be unblinded.

The population will be stratified with regard to
- ASA-class (2 strata: ASA≤2 and ASA=3) and
- investigational centre

Stratification variables will be included as covariates in the primary analysis (according to CPMP/EWP/2863/99).

10 INVESTIGATIONAL PRODUCTS

Appropriate amount of the Investigational Products will be made available to hospital pharmacist by B. Braun Melsungen AG. If defects in the Investigational Products are observed, the study manager or the monitor is to be informed.

10.1 Name and Description of the Investigational Product(s)

10.1.1 Qualitative and Quantitative Composition

The following table presents the composition of the Investigational Products, i.e.

1000 ml solution contains:

	**Investigational Test Product**	**Investigational Reference Product**	**Descriptive Control Investigational Reference Product**
	Tetraspan 10%	Tetraspan 6%	Sterofundin ISO
	hyper-oncotic solution of HES 130/0.42/6:1 in plasma adapted Ringer's solution (balanced solution)	iso-oncotic solution of HES 130/0.42/6:1 in plasma adapted Ringer's solution (balanced solution)	plasma adapted Ringer's solution (balanced electrolyte (crystalloid) solution)
**Ingredients/ per 1L**			
HES 130/0.42	100 g	60 g	--
sodium chloride	6.25 g	6.25 g	6.80 g
potassium chloride	0.30 g	0.30 g	0.30 g
calcium chloride dihydrate	0.37 g	0.37 g	0.37 g
magnesium chloride hexahydrate	0.20 g	0.20 g	0.20 g
sodium acetate trihydrate	3.27 g	3.27 g	3.27 g
malic acid	0.67 g	0.67 g	0.67 g
**Electrolyte concentrations per 1L**			
sodium	140.0 mmol	140.0 mmol	140.0 mmol<br>145.0 mmol*
potassium	4.0 mmol	4.0 mmol	4.0 mmol
calcium	2.5 mmol	2.5 mmol	2.5 mmol
magnesium	1.0 mmol	1.0 mmol	1.0 mmol
Chloride	118.0 mmol	118.0 mmol	127.0 mmol/l
acetate	24.0 mmol	24.0 mmol	24.0 mmol
malic acid	5.0 mmol	5.0 mmol	5.0 mmol
**Further characteristics**			
pH	5.6 – 6.4	5.6 – 6.4	5.1 – 5.9
theoretical osmolarity	297 mOsmol/l	297 mOsmol/l	309 mOsmol/l
titration acidity	$<$ 2.0 mmol/l	$<$ 2.0 mmol/l	~ 5 mmol/l

For HES an inhouse-specification applies while all other ingredients are in accordance to the European Pharmacopoeia (Ph. Eur.).

The Investigational Products will be manufactured and released by the Sponsor B. Braun Melsungen AG, Melsungen, Germany in compliance with Good Manufacturing Practice (GMP). For batch number and expiry date see certificate of analysis.

10.1.2 Pharmaceutical Form

Clear colourless aqueous solution for infusion

10.1.3 Nature and Content of Container(s)

Polyethylene plastic bottle (Ecoflac plus®), 500 ml

Ready-to-use container for single use only. The Investigational Products should be administered immediately after connecting the container to the giving set.

10.2 Posology and Method of Administration

10.2.1 Dosage

Administration of Investigational Test and Reference Products will be performed as an “add on” fluid administration to a continuous basal crystalloid infusion.

The continuous basal infusion of the crystalloid will be 4 ml/kg bodyweight/h. A balanced crystalloid will be used for basal infusion (Sterofundin ISO).

The “add on” fluid administration of the Investigational Products i. e. of either HES 10%, HES 6% or crystalloid in a blinded manner with boluses of 250 ml will individually be controlled by measuring stroke volume with oesophageal Doppler to achieve a target controlled preload optimum. A detailed algorithm (flow diagram) will provide basis for amount of volume replacement (see also appendix 6)

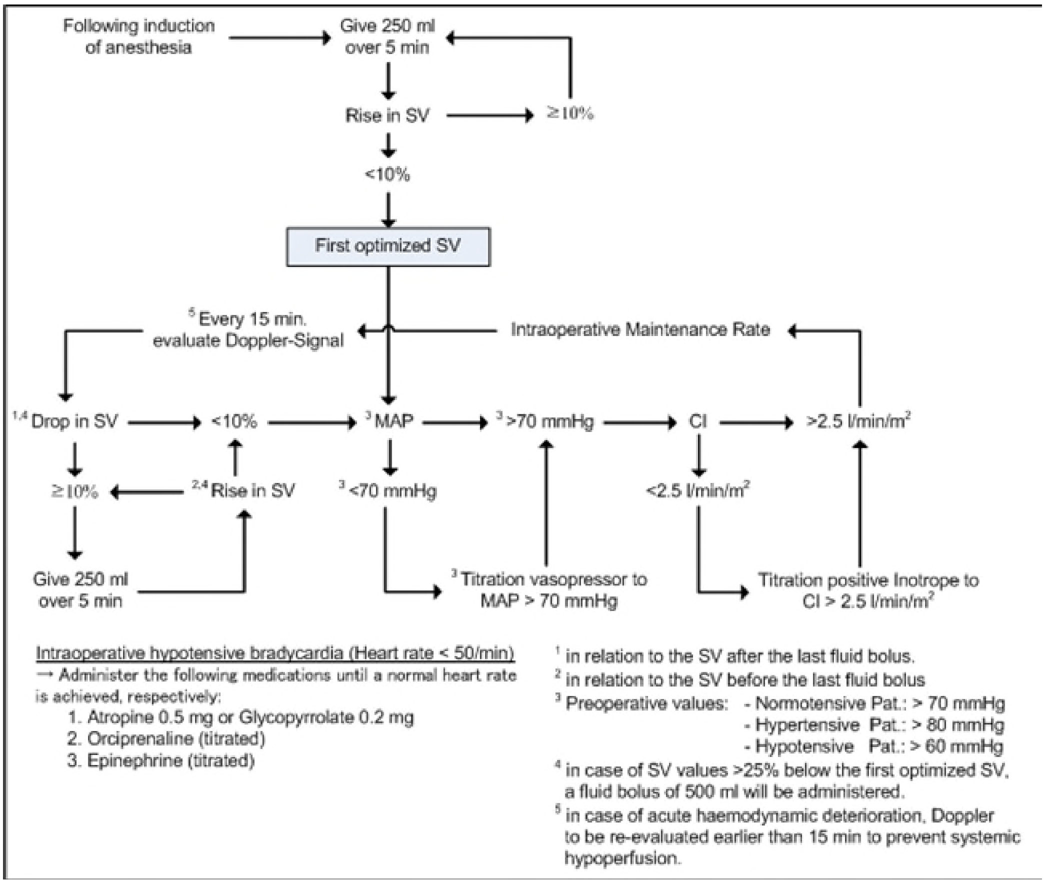

If during surgery an acute haemodynamic deterioration occurs, indicated by an increase in heart rate and a concomitant drop in arterial blood or central venous pressure (as it might occur due to a surgical cause of bleeding) then the re-evaluation

of the Doppler signal has to be performed earlier than after 15 minutes to prevent systemic hypoperfusion.

In case intra-operative volume requirements exceed the amount of bottles of Investigational Product supplied by the pharmacy in a blinded manner then open-label treatment with a balanced crystalloid solution (Sterofundin ISO) will be performed during surgery (also using oesophageal Doppler measurements for deciding on the administration of the fluids).

10.2.2 Method of Administration

The administration of the Investigational Products is performed intravenously.
Do not mix with other medicinal products.

10.2.3 Duration of treatment

Treatment with Investigational Product starts after reliable basal oesophageal Doppler measurements are obtained and before skin incision.

After final positioning of the patient in the operating theatre the oesophageal probe will be placed and basal measurements will be recorded after correct placement of probe and reliable measures are obtained. Thereafter, before skin incision, the first bolus of Investigational Product of 250 ml/5 min will be given and the algorithm of the individualized volume replacement therapy is initiated. Oesophageal Doppler guided volume replacement will be performed throughout surgery.

Treatment with Investigational Product ends with surgery when the oesophageal probe is withdrawn. In case all bottles of Investigational Product as compiled by the pharmacist are used up before end of surgery then open-label treatment (in addition to basal balanced crystalloid infusion) with a balanced crystalloid (Sterofundin ISO) will be performed from then on during surgery.

After surgery, on ICU, volume replacement will be performed at the discretion of the attending physician. In case colloidal volume replacement is required on ICU on the day of surgery no HES-product but other colloidal volume replacement (e. g. gelatine) will be used. All volume/fluid replacement therapy will be recorded (separately for colloids and crystalloids).

(Note: The loading of the epidural catheter and start of continuous epidural analgesia will be performed at the end of surgery after all intraoperative, study-related measurements have been finalized.)

10.3 Labelling

The label will be designed in accordance with GMP annex 13 and will contain the information as required by the pertinent national requirements.

See below example for label on the primary container.

As the Investigational Products will be supplied as bulkware to the hospital's pharmacy the secondary label of the respective cartons will only indicate the sponsor's name B. Braun Melsungen AG and the content (i. e. the respective product Tetraspan 10%, Tetraspan 6% or Sterofundin ISO) with batch number and expiry date and the amount of bottles in the carton.

**Nur zur klinischen Prüfung bestimmt**<br>Studien-Nr.:<br>HC-G-H-0803<br>EudraCT-Nr.:<br>2008-004175-22<br>balanziertes Volumenersatzmittel<br>(kolloidal oder kristalloid)<br>500 ml Infusionslösung zur intravenösen Anwendung<br>Random Nr.<br>|__|__|__|__|__| Flaschen-Nr.<br>|___|___| **Flaschen nur in aufsteigender Reihenfolge verwenden!**<br>Gesamtzahl der Flaschen für diesen Patienten<br>|__|__| Hauptprüfarzt:	Anwendung und Dosierung gemäß Studienprotokoll<br>Nur verwenden wenn die Lösung klar und frei von Partikeln und die Verpackung nicht beschädigt ist.<br>Nach Öffnen der Primär-verpackung sofort verwenden<br>Zur einmaligen Anwendung; etwaige Reste verwerfen<br>Nicht über 25°C lagern, nicht im Kühlschrank lagern und nicht einfrieren.<br>Flasche nach der Behandlung aufbewahren. und zur Zentrumsapotheke zurückschicken.<br>Code Nummer:<br>Verwendbar bis MM/YYYY<br>Ident.-Nr.:<br>B|BRAUN<br>**B. Braun Melsungen AG**<br>Carl-Braun-Str. 1<br>34212 Melsungen<br>Tel.: 05661-71-0

Further Principal investigator (Hauptprüfarzt) to be mentioned on the respective labels:

centre Berlin:

centre Bonn

10.4 Packaging

The hospital's pharmacy will be supplied with bulk ware of the Investigational Products with labels of the primary container as stated above. This will enable the pharmacist to compile individual amounts (units/bottles) of Investigational Products as described in detail in section 9.5.

10.4.1 Storage

The Investigational Products will be stored separately in the hospital's pharmacy. The pharmacy will supply the Investigational Products in time as requested by the investigator and according to randomisation and blinding procedures (see section 9.5).

Do not freeze, do not store in refrigerator, do not store above 25°C.

10.4.2 Investigational Product Accountability

In accordance with regulations, the pharmacist or other appropriate individual, who is designated by the Investigator, will keep an inventory of all clinical trial material (CTM) received. All used and unused CTM must be accounted for on an Inventory Form which will be provided to the pharmacist by the Site Monitor (appendix 7). CTM inventory forms will be examined and reconciled by the Investigator at the end of the study.

These records should include dates, quantities, batch / serial numbers, expiration dates (if applicable), and - if applicable - the unique code numbers assigned to the Investigational Product(s) and study patients. Investigators should maintain records that document adequately that the patients were provided the doses / treatments specified by the protocol.

At the end of surgery all bottles i. e. used (empty or with remainder) as well as unused/unopened bottles which have been compiled by the pharmacist for the individual patient must be returned to the pharmacist for product accountability.

A copy of the completed Inventory Form must be retained in the Investigator's Site File, and a copy must be filed in the Sponsor Trial Master File.

10.5 Destruction / Retrieval of Surplus Investigational Products

Surplus Investigational Products are to be returned to the Sponsor after termination of the entire study. The Sponsor is responsible for the destruction of unused Investigational Products.

11 CONCOMITANT THERAPY

Preoperative medication (e. g. ß-blockers, statins, ACE-I, antidiabetics) will be documented on the CRF.

Duration of preop fasting time is standardized (solid food on demand of the attending surgeon, clear fluids up to 2 hours preop) and will be documented in the CRF. Furthermore, type of bowel preparation will be documented in the CRF.

Preoperative medication will be continued on the day of surgery with the exception of subcutaneous rapid-onset insulin, metformin, acetylsalicylacid and MAO monoamine oxidase)-inhibitors.

Anaesthetic technique will follow a standardized protocol and usually is based on a volatile anaestethic using desflurane.

Epidural analgesia using ropivacaine 0.2% and sufentanil (concentration will be left to the standard operating procedures of the study centres) 1 mcg/mL will be started at the end of surgery after all study measurements have been completed.

Necessary concomitant medication during anaesthesia and pancreatic surgery:

- Basal balanced crystalloid (Sterofundin ISO) infusion of 4 ml/kg BW/h.
- Inotropic drugs (e. g. noradrenalin, dobutamin, adrenalin, orciprenalin, atropine, enoximone, cafedrin-theodrenalin, levosimendan) will be applied when required and as deemed necessary by the treating physician.
- Administration of sodium bicarbonate 8.4% if pH<7.25 and/or base excess>-10 mmol/l
- Transfusion of erythrocyte contentrate (EC): Trigger is an haematocrit of < 25%. In case severe bleeding occurs EC and fresh frozen plasma (FFP) may be given independently from trigger according to the physician's judgement.
- It is aimed at to transfuse fresh frozen plasma (FFP): following a proportion of EC:FFP = 2:1 for the first 4 infusions of EC; after 4 infusions of EC the proportion is 1:1.
- Infusion of platelet concentrate when thrombocyte count is < 50,000/ µl

- If volume requirement during surgery exceeds the amount of Investigational Product compiled then open label fluid replacement with a balanced crystalloid (Sterofundin ISO) is performed.

Post-operative patient management

A standardised therapy management with respect to enteral nutrition, mobilisation etc will be applied and documented in detail in the CRF.

On ICU volume replacement will be performed at the discretion of the attending physician. See “unauthorised concomitant medication”.

For the assessment of the second primary variable, time until fully on oral diet, it has to be assessed for a period of maximally 15 days. It will be assessed for each day (on the subsequent day) whether the patient receives parenteral nutrition. As soon as the patient is off parenteral nutrition and has taken nutrients via oral (not enteral) pathway at least three times that day no further recording of nutrional diet is required.

Unauthorised concomitant medication:

Aside from basal fluid administration no other colloid or crystalloid volume replacement agents than study medications will be applied *during surgery* as long as Investigational Product as compiled by the pharmacist is available, with the exception of concentrated electrolytes for individual corrections.

On ICU if volume replacement with colloidal is required on the day of surgery no HES-product but other colloidal solutions (e. g. gelatine) will be used.

12 DEFINITION OF THE PRIMARY AND SECONDARY VARIABLES

12.1 Primary Variable

First primay endpoint: Intraoperatively required amount of HES (mLs) under target controlled volume replacement therapy.

Second primary endpoint: Time until fully on oral (solid) diet (days): Fully on oral (solid) diet is reached when no more parenteral and enteral nutrition is given and the patient has taken nutrients via the oral pathway for at least three times that day.

12.2 Secondary Variables - Safety

<u>Haemodynamics</u>

- SAP, DAP, MAP, HR, CVP
- CI, SV, SVR using oesophageal Doppler and PiCCO
- cumulative dose of norepinephrine (mcg/h)
- intrathoracic blood volume index (ITBVI) and extravascular lung water index (EVLWI) (PICCO)

Measurements
of SAP, DAP, MAP, HR, CVP

- baseline
- intraoperatively according to dosing algorithm cycle: *i.e. in 15 min intervals when no volume optimisation, or 15 minutes after new optimised stroke volume*
- at the end of surgery

- on ICU every 6 hours

of CI, SV and SVR (oesophageal Doppler and PiCCO)
- baseline
- intraoperatively according to dosing algorithm cycle: *i.e. in 15 min intervals when no volume optimisation, or 15 minutes after new optimised stroke volume*
- at the end of surgery
- PiCCO only: on admission to ICU, 6 hours after admission, from post-op day +1 every 12 hours (morning and evening)

of ITBI and EVLWI (PiCCO)
- baseline
- every 60 minutes after start of surgery
- end of surgery
- on ICU at admission and 6 hours thereafter, from 1st post-op day on 12-hourly (morning and evening)

Blood gas analysis and acid-base status, electrolytes

- pCO2, pO2, HCO3, SaO2
- pH, base excess, bicarbonate, lactate
- sodium, potassium, ionized calcium, chloride

Blood will be sampled as close as possible to the time, at which the corresponding hemodynamic measurement is performed. Measurements will be at
- baseline: arterial blood sample
- intraoperatively (every 60 minutes), arterial blood sample
- postoperative (at the end of surgery): arterial blood sample
- on ICU (including postoperative day 1) on admission, 6 hours after admission, on POD 1 every 6 hours venous or arterial blood gas sample

Postoperative inflammatory reaction

- IL6 (facultative), IL10 (facultative), PCT, CRP
- SIRS-criteria (body temperature, leucocytes, HR, pCO2)

Measurements will be
facultative for IL6, IL10
- baseline
- end of surgery
- 1st postoperative day in the morning

PCT, CRP
- baseline
- 1st postoperative day in the morning

SIRS-criteria
- 1st postoperative day in the morning
- on ICU once daily (in the morning)

Renal function

- diuresis/urinary output
- Serum: creatinine, BUN (calculated from serum urea measurement), HbA1c
- Urine: creatinine, α1-Mikroglobulin (α1-MG), N-Acetyl-ß-D-Glucosaminidase (ß-NAG)

Measurements will be
Diuresis
- intraoperatively cumulative every 60 min
- on ICU 24 hours period

BUN, ß-NAG, α1-MG
- baseline
- 1st postoperative day in the morning
- on ICU once daily in the morning

Creatinine
- baseline
- 1st postoperative day in the morning
- on ICU once daily in the morning
- serum creatinine: postoperative days 3 and 7 in the morning

HbA1c
- baseline

Haemostasis

- PT, aPTT, Fibrinogen
- vWF-Ag, vWF-Ristocetin-Cofaktor (vWF-RiCo),
- FVIII:c,
- ROTEM derived parameters: CT, CFT, MCF (ExTEM and FibTEM)

Measurements will be
- baseline
- intraoperatively: 1h after start of operation (ROTEM only)
- at the end of surgery
- 1st postoperative day in the morning (PT, aPTT, vWF-Ag, vWF-RiCo, ROTEM)

Cardiac function

- Troponin or high-sensitivity Troponin
- NT-Pro-BNP

Measurements will be
- baseline
- end of surgery
- 1st postoperative day

Other

- PONV (postoperative nausea and/or vomiting)
- Adverse events will be documented with start of administration of Investigational product throughout surgery and postoperatively on ICU until postoperative day 5 (including). Serious adverse events that are not yet resolved at the POD 5 will be followed up until study end.

Circulating blood volume (mLs): Calculation will use the following formulas:
- males: ((0.3669 x height (m) + 0.03219 x weight (kg) + 0.6041) x 1000
- females: ((0.3061 x height (m) + 0.03308 x weight (kg) + 0.1833) x 1000
 (Nadler S, Surgery 1962, Mercuriali F, Curr Med Res Opin 1996).
- baseline
- 1st postoperative day (if weight measurement can be performed)

Requirements of blood products (EC, FFP, PC, plasma derived coagulation factor concentrates) and intraoperative blood loss (suction volume)

NOTE: measurements on ICU (if not otherwise mentioned) will be performed until "fit for discharge criteria are fulfilled or until day +5 (incl. day +5) whatever occurs first. Adverse events and adverse reactions will be recorded until post-op day +5. At three months after surgery the patient will be interviewed for health related quality of life (HRQoL).

12.3 Secondary Variables - Efficacy

Haemodynamics

- SAP, DAP, MAP, HR, CVP, CI, SV, SVR
- cumulative dose of norepinephrine (mcg/h)
- intrathoracic blood volume index (ITBVI) and extravascular lung water index (EVLWI), (PICCO)

Measurements see 12.2

Ventilatory support

- Time on ventilator (measured from intubation in hours)
- Requirements of postoperative ventilatory support (PCV, CPAP)

Nursing Delirium Screening Scale (NuDesc) (see appendix 8)

Lütz A et al. (2008)

includes 5 items, ratings 0 – 2

Measurements will be

on ICU three times daily

Injury scores (see appendix 8)

- SOFA
  Vincent et al., (1996)
- SAPS II
  Le Gall JR et al. (1993)
- APACHE II
  Knaus et al. (1985)

Measurements
on ICU: once daily

Gastrointestinal parameters

- bowel movements on auscultation
- time to full oral nutrition
- time to first defaecation
- time to first flatus

Intraabdominal pressure

Measurements will be
- baseline (after induction of anaesthesia)
- at the end of surgery (still in operating theatre, directly before extubation)
- 6 hours after end of surgery
- 1st post-operative day in the morning

Technique:
Intraabdominal pressure will be obtained by measuring the intravesical pressure with the patient in a supine position. The bladder will be flushed via the transurethral catheter via a three-way stopcock using 25 mLs of sterile saline. The bladder will be emptied until air reaches three-way stoppcock in order to exclude air bubbles in the system and to guarantee patency. As measurement volume 25 mLs sterile saline will be instilled. The manometer/pressure transducer will be connected to the transurethral catheter/connection to bladder via a three-way stopcock. Zero-reference will be at the midaxillary axis (same reference as CVP). Confirmation of correct measurement will be done by control of respiratory variation and the endexpiratory value will be noted.

In the awake patient (measurement at 6 hours after end of surgery and on 1st postoperative day in the morning) pain assessment will be made prior measurement of intraabdominal pressure using visual analogue scale (VAS).

Categories for pain assessment will be

VAS 0	no pain
VAS 1-3	mild pain
VAS 4-6	medium/moderate pain
VAS 7-10	intense pain

Mobilisation

Time until fit for discharge criteria (Marshall S. I., F. Chung (1997, 1999) (appendix 8) are fulfilled.

Other

Aldrete Score (refer to in Marshall et al. 1999, appendix 8): on admission to ICU and once daily on ICU.

Length of stay in ICU/IMCU (fulfillment of discharge criteria) – assessment will be done once daily (evening) on ICU. If fit-for discharge criteria are not yet fulfilled at POD 5 it will be continued to check until fulfillment or until study end, whatever orccurs first.

Morbidity and complication rate: surgical complications such as re-do surgery (e. g. anastomotic insufficiency or bowel leakage), disturbed wound-healing or medical complications which delay time to fully oral nutrition will be assessed and documented at study end

Combined outcome parameter of prolonged length of stay (based on mean length of stay according to the German Diagnosis Related Group System D-DRG) or death.

Health Related Quality of Life (HRQoL) before and 3 months after surgery (EQ-5D, the EuroQol Group, http://www.euroqol.org)

NOTE: measurements on ICU (if not otherwise mentioned) will be performed until "fit for discharge criteria are fulfilled or until day +5 (incl. day +5) whatever occurs first.

12.4 Other variables

Demographic data (age, gender, weight, body mass index (BMI))

Surgery related data (duration of surgery and anesthesia, precise classification of surgical procedure)

Concomitant medication/therapy on ICU (see NOTE above)

12.5 Source Documents

For definitions of source data and source documents see 17.1.

The following table represents the source documents for the data to be entered in the CRF:

Variable	Source document
Informed consent	Informed consent form, patient chart
Patient's data (demographics: sex, age, indication, concomitant diseases, history etc.)	Patient chart (ethnicity CRF)
Exclusion criteria that refer specifically to the placement of the Doppler tube	Study specific working sheet
ASA	CRF
Weight (measured)	CRF
Pre-operative measures (questioning of patient)	CRF
IAD	CRF
Indication	Anaesthesia chart
Description of procedure	CRF
Investigational Product administration	Intraoperative and postoperative charts, study specific working sheet
Concomitant medication	Intraoperative and postoperative charts
Haemodynamics	Intraoperative and postoperative charts, study specific working sheets
Laboratory measures	Laboratory print-out
Aldrete Score, NuDesc, fit-for-discharge criteria	CRF
PONV	CRF
Nutrition intake (questioning of patient) and gastrointestinal parameters (time	CRF

Variable	Source document
until first flatus, time until full oral nutrition, first bowel movement, time until first defaecation	
Adverse events	Intraoperative and postoperative charts, patient chart<br>CRF
HRQoL	Questionaire
Final assessment	CRF
Study termination	CRF, patient chart

13 ASSESSING AND REPORTING OF ADVERSE EVENTS

Throughout the course of the clinical trial particular attention is paid to Adverse Events and Adverse Drug Reactions.

13.1 Definitions

13.1.1 Adverse Events

An Adverse Event (AE) is any untoward medical occurrence in a patient or clinical investigation subject administered a pharmaceutical product and which does not necessarily have a causal relationship with this treatment. An AE can therefore be any unfavourable and unintended sign (including an abnormal laboratory finding), symptom, or disease temporally associated with the use of an Investigational Product, whether or not related to the Investigational Product or study treatment/procedures.

13.1.2 Adverse Reaction

Adverse Reactions (AR) are all untoward and unintended responses to an Investigational Product related to any application / dose administered. All adverse events judged by either the reporting investigator or the sponsor as having a reasonable causal relationship to a medicinal product qualify as AR. The expression reasonable causal relationship means to convey in general that there is evidence or argument to suggest a causal relationship.

Regarding marketed Investigational Products: a response to a product which is noxious and unintended and which occurs at applications normally used in man for prophylaxis, diagnosis, or therapy of diseases or for modification of physiological function.

13.1.3 Unexpected Adverse Reactions

An AR, the nature or severity of which is not consistent with the applicable product information (= reference document, e. g. Investigator's Brochure (IB) for an unauthorised Investigational Product or SmPC for an authorised product).

When the outcome of the AR is not consistent with the applicable product information this AR should be considered as unexpected.

The applicable product information/reference document in the present study is the SmPC as identified in section 19.9.

13.1.4 Serious Adverse Event (SAE) or Serious Adverse Reaction (SAR)

Any untoward medical occurrence or effect that at any dose

- Results in Death
- Is life threatening
- Requires hospitalisation or prolongation of existing inpatients' hospitalisation
- Results in persistent or significant disability or incapacity
- Is a congenital anomaly or birth defect

More than one of the above criteria can be applicable to the one event.

NOTE:

Death: is the outcome of an SAE. The event to be reported comprehensively is the medical condition leading to death, e.g. underlying disease, accident.

Life-threatening: in the definition of a SAE or SAR refers to an event in which the patient was at risk of death at the time of the event; it does not refer to an event which hypothetically might have caused death if it was more severe.

Hospitalisation is defined as inpatient care of more than one calendar day (overnight admission). Admission for ambulant diagnostic procedures, overnight survey visits or ambulant visits to an emergency ward, e. g. during weekends are not considered 'hospitalisation' in the sense of the criteria for SAE / SAR, unless any of the other criteria for serious is met.

Medical judgement should be exercised in deciding whether an AE / AR is serious in other situations. Important: AEs / ARs that are not immediately life-threatening or do not result in death or hospitalisation but may jeopardise the patient or may require intervention to prevent one of the other outcomes listed in the definition above, should also be considered serious.

13.1.5 Adverse Event Intensity

Intensity	Definition
Mild	Patient is aware of signs and symptoms but they are easily tolerated
Moderate	Signs / symptoms cause sufficient discomfort to interfere with usual activities
Severe	Patient is incapable to work or perform usual activities

Cave: The term "severe" is often used to describe the intensity (severity) of a specific event. This is not the same as 'serious', which is based on patient/event outcome or action criteria (see definition 11.1.4)!

13.1.6 Adverse Event Causality

Causality code	Definition
Not assessable	A report suggesting an AE, which cannot be judged because information is insufficient or contradictory, and which cannot be

	supplemented or verified.
Unlikely	A clinical event, including laboratory test abnormality, with a temporal relationship, which makes a causal relationship improbable, and in which other drugs / treatments, chemicals or underlying disease(s) provide plausible explanations.
Possible	A clinical event, including laboratory test abnormality, with a reasonable temporal relationship to administration of the drug / treatment, but which also could be explained by concomitant diseases or other drugs / treatments or chemicals.
Probable	A clinical event, including laboratory test abnormality, with a reasonable temporal relationship to administration of the drug / treatment, unlikely to be attributable to concomitant disease(s) or other drugs / treatments or chemicals, and which follows a clinically reasonable response on withdrawal (dechallenge). Rechallenge information is not required to fulfil this definition.
Certain	A clinical event, including laboratory test abnormality, occurring in a plausible time relationship to study treatment and which cannot be explained by concomitant disease(s), other drugs / treatments or chemicals. The response to withdrawal of the treatment (dechallenge) should be clinically plausible. The event must be unambiguously either pharmacologically or as phenomenon, using in satisfactory rechallenge procedures if necessary.

13.2 Recording and Reporting Adverse Events and Adverse Reactions

13.2.1 Recording

The Investigator must record in detail all AEs (signs and symptoms) which are either volunteered by patients or observed during or following the course of Investigational Product administration and during the course of the study on the appropriate CRF page.

Included in the description should be

- the nature of the sign or symptom;
- the date of onset; date of resolution (duration);
- the severity / intensity (for definition see section 13.1.5);
- the investigator’s judgement on possible relationship to study treatment or other therapy (for definition see section 13.1.6);
- the action taken (if any), and
- the outcome.

During surgery and during stay on ICU several events including deviation from normal laboratory values occur which are events due to underlying disease and treatment and are therefore, not considered to be adverse events. This includes e. g. the specified use of inotropes in order to keep the patient hemodynamically stable, the use of specified amount of blood products in order to replace surgically caused blood loss, specified insulin treatment for blood sugar control on ICU, pre-specified increase in body temperature within a specified time period after surgery, etc. The pre-specified events, laboratory deviations respectively time points and periods are listed in detail in appendix 10 . This list is to be used in order to harmonize adverse event reporting during this multicentric study. Nevertheless, concomitant medication and reason for it will be documented as described in section 11.

13.2.2 Reporting of Serious Adverse Events and Unexpected Adverse Reactions

All SAEs (for exemptions see below), whether or not deemed Investigational Product-related or expected must be reported to the Sponsor by telephone within 24 hours (one working day) of the Investigator becoming first knowledge:

Contact Name: see "Responbilities and Addresses (section 1)

A written report must follow within five working days and is to include a full description of the event and sequelae, in the format detailed by the Serious Adverse Event reporting form provided by the Sponsor (see appendix 9).

The Sponsor will notify the competent authorities, IECs and all investigators concerned of suspected ARs which are unexpected (SUSARs) in line with pertinent legal requirements.

While reporting adverse events, all pertinent data protection legislation must be adhered to.

<u>Exemptions from expedited reporting:</u> SAEs that are known for the indication respectively the surgery have not to be reported on an expedited basis but will be fully documented on the AE-CRF-page. In detail, the following SAEs are exempted from expedited reporting: Anastomotic insufficiency, Re-intubation, re-do surgery, (not bleeding), postoperative delir.

13.3 Adverse Event follow-up procedures

Adverse Events will be followed up throughout the course of the clinical trial.

13.4 Potential Risks and Potential Adverse Events

As proposed treatment is in line with normal procedures in elective pancreatic surgery no additional risk is considered to arise from the present study.

According to the SmPCs for Tetraspan (10% and 6%) and for Sterofundin ISO the following adverse drug reactions are described:

Tetraspan: The most common side effects observed are directly related to the therapeutic effect of starch solutions and the doses given, i.e. dilution of the blood as a result of the filling of the intravascular space without administering blood components at the same time. Coagulation factor dilution can also occur.

Sterofundin ISO: Overdose or too fast administration may lead to water and electrolyte (including acetate, malate) overload with respective signs and symptoms as described in SmPC section overdose.

<u>Blood and lymphatic system disorders</u>
Tetraspan: Very common: Decreased haematocrit and reduced concentration of plasma proteins due to dilution. Common (depending on the administered dose): Relatively large doses of hydroxyethyl starch result in dilution of coagulation factors and can therefore affect blood coagulation. Bleeding time and aPTT can be prolonged and the level of FVIII/vWF complex can be reduced after administration of large doses.

<u>Immune system disorders</u>
Tetraspan: Rare: Anaphylactoid reactions of varying degrees. Hypersensitivity reactions which occur very rarely are not dose-dependent.

Sterofundin ISO: Occasionally: Hypersensitivity reactions characterized by urticaria have been occasionally described after the intravenous administration of magnesium salts.

Gastrointestinal disorders

Sterofundin ISO: Rare: Although oral magnesium salts stimulate peristalsis, paralytic ileus has been rarely reported after intravenous infusion of magnesium sulphate.

General disorders and administration site conditions
Tetraspan: Uncommon: Repeated infusions of HES for several days, particularly when high cumulative doses are reached, generally result in itching which responds poorly to any therapy. This itching can occur several weeks after the end of the starch infusions and can persist for months. The probability of this undesirable effect has not been sufficiently studied with Tetraspan10% and Tetraspan 6%

Sterofundin ISO: Adverse reactions may be associated to the technique of administration including febrile response, infection at the site of injection, local pain or reaction, vein irritation, venous thrombosis or phlebitis extending from the site of injection and extravasation.

Laboratory investigations
Tetraspan: Very common: Infusion of hydroxyethyl starch results in increased serum alpha-amylase levels. This effect is a result of the formation of an amylase complex of hydroxyethyl starch with delayed renal and extrarenal elimination. This should not been misinterpreted as evidence of a pancreatic disorder.

13.5 Pregnancies

If a female patients gets pregnant while participating in the present study this event has to be recorded and reported using the Serious Adverse Event Reporting Form (template see annex 8). If possible outcome of birth should be recorded.

14 VISIT SCHEDULE

14.1 Procedures at Each Visit

day -1(range -5)/ pre-operatively:

- Verification of inclusion and exclusion criteria*, including informed consent
- History, demographics, body weight
- Randomization
- HRQoL (EQ-5D questionnaire)
- Pre-operative medication / therapy as medically indicated and standardized (including pre-operative fasting time, type of bowel preparation)
  - Pre-op transfusion will be administered according to standard procedure (hct. between 25 and 30%)

* blood samples for laboratory evaluation of haematocrit, renal function/serum creatinine, hepatic function/Quick value, Child-Pugh, electrolyte status/ Na, K, Ca

* urine sample in women: for pregnancy testing

day 0/ prior anaesthesia:

- Start basal infusion of crystalloid fluid replacement: 4 ml/kg/h
- Insert thoracic epidural catheter
- Induction anaesthesia
- Adverse events

day 0/ after induction of anaesthesia/prior surgery :

- Set arterial line and central venous catheter
- After set of urethral catheter measurement of intraabdominal pressure
- Final positioning of patient in operating theatre
- Placement of oesophageal probe, calibration of PiCCO
- Baseline recording of haemodynamics (SAP, DAP, MAP, HR, CVP; oesophageal Doppler and PiCCO: CI, SV, SVR; PiCCO: ITBVI, EVLWI)
- Body temperature
- Blood sampling and urine sample for laboratory evaluation*
- Concomitant medication and therapy (including concomitant medication prior induction of anaesthesia; including blood products acc. to trigger)
  Note: Loading dose of epidural analgesia will be applied at the end of surgery after all intraoperative study related measurements have been finalized
- Adverse events

Baseline laboratory

- o arterial blood gas analysis (pCO2, pO2, HCO3, SaO2) including acid base status (pH, BE, lactate, bicarbonate) and electrolytes (Na, K, Ca, Cl). Blood sampling for BGA should be performed as close as possible to the time, at which the corresponding hemodynamic measurement is done.
- o renal function parameters (*urine*: creatinine, α1-MG, β-NAG; *serum*: creatinine, BUN, HbA1c)
- o haemostasis and coagulation parameter (PT, aPTT, Fibrinogen, vWF-Ag, vWF-RiCo, F VIII:c; CT, CFT, MCF using ExTEM und FibTEM)
- o Inflammamatory parameters (IL6 (facultative), IL 10 (facultative), PCT, CRP, white blood cell count (WBC))
- o cardiac function parameters (Troponin or high-sensitivity Troponin, NT-Pro-BNP)

day 0/ after baseline recordings:

- First bolus (250 mLs/5 min) of Investigational Products
- Start of volume replacement algorithm according to oesophageal Doppler measurements
- Thereafter: start of surgery/skin incision
- Adverse events and adverse reactions

day 0/ during surgery (after start of skin incision):

- Haemodynamics
  - o every 15 min within dosing algorithm cycle when there is no volume optimisation, or 15 minutes after stroke volume optimisation: SAP, DAP, MAP, HR, CVP; PiCCO and oesophageal Doppler: CI, SV, SVR
  - o every 60 min after start of surgery: ITBVI, EVLWI (PiCCO)
- Infusion of the Investigational Products according to algorithm
- Hourly blood sampling for intraoperative laboratory evaluation*
- Diuresis/urinary output (cumulative every 60 min)
- Concomitant medication and therapy (including inotropic drugs, sodium bicarbonate, infusion of blood products (RBC, FFP, platelets) according to the triggers
- Adverse events and adverse reactions

*laboratory measurements in blood/serum samples:

- o 1 hour after start of surgery: haemostasis/ coagulation (ROTEM only), arterial blood gas analysis (pCO2, pO2, HCO3, SaO2) including acid base status (pH, BE, lactate, bicarbonate) and electrolytes (Na, K, Ca, Cl)
- o at 2 hours after start of surgery and then every 60 min intraoperatively: arterial blood gas analysis (pCO2, pO2, HCO3, SaO2) including acid base status (pH, BE, lactate, bicarbonate) and electrolytes (Na, K, Ca, Cl)
- o blood sampling for BGA should be performed as close as possible to the time, at which the corresponding hemodynamic measurement is done. i.e. ITBVI and EVLWI.

day 0/ at the end of surgery:

- Haemodynamics (SAP, DAP, MAP, HR, CVP; PiCCO and oesophageal Doppler: CI, SV, SVR; PiCCO: ITBVI, EVLWI)
- intraabdominal pressure (still in operating theatre directly before extubation)
- Concomitant medication and therapy, blood products
- Blood sampling for laboratory evaluation*
- Investigational Products
- Diuresis/urinary output
- Adverse events and adverse reactions
- Loading dose of epidural analgesia will be applied at the end of surgery after all intraoperative study related measurements have been finalized

* laboratory measurements in blood/serum sample:

- o arterial blood gas analysis/acid base status/electrolytes. Blood sampling for BGA should be performed as close as possible to the time, at which the corresponding hemodynamic measurement is done.
- o haemostasis/coagulation parameters (PT, aPTT, Fibrinogen, FVIII:c, vWF-Ag, vWF-RiCo; CT, CFT, MCF using ExTEM und FibTEM)
- o cardiac function (Troponin or high-sensitivity Troponin, NT-Pro-BNP)
- o inflammatory reaction parameters (facultative: IL-6, IL-10)

day 0/ intensive care unit (ICU) –

- Aldrete Score (*on admission)*
- Haemodynamics (SAP, DAP, MAP, HR, CVP (on admission and 6hourly); PiCCO: CI, SV, SVR, ITBVI, EVLWI) (on admission and after *6 hours*)
- blood gas analysis (arterial or venous blood sample) including acid base status and electrolytes: (on admission, 6 hours after admission). Blood sampling for BGA should be performed as close as possible to the time, at which the corresponding hemodynamic measurement is done.
- abdominal pressure *(6 hours after admission)*
- Ventilatory support as requested to maintain normocapnia and oxygen saturation (PCV, CPAP)
- Concomitant medication, infusion therapy (separate for colloids (i. e. gelatine) crystalloids, blood products)
- Diuresis (sampling period until next morning about 6 o'clock)
- Adverse events and adverse reactions

- check of discharge criteria

day +1 (1st post-operative day)/ On ICU: Postoperative care will follow a standardised regimen.

- Haemodynamics (SAP, DAP, MAP, HR, CVP(every 6 hours)); PiCCO: CI, SV, SVR, ITBVI, EVLWI (every 12 hours/ in the morning and in the evening)
- abdominal pressure (in the morning)
- Ventilatory support (PCV, CPAP) as requested to maintain normocapnia and oxygen saturation
- Blood sampling for laboratory evaluation*, urine sample for laboratory evaluation**
- Aldrete Score (in the morning)
- SIRS-criteria
- Nursing Delirium Screening Scale (NuDesc) (three times daily)
- Injury scores (APACHE II, SAPS II, SOFA)
- Assessment of “fit for discharge” criteria (once on ICU - preferably in the morning)
- Concomitant medication including parenteral nutrition, infusion therapy (separate for colloids i. e. gelatine and crystalloid, blood products)
- Diuresis (Sampling period is from morning until next morning about 6 o’clock)
- PONV
- body weight (if possible)
- Gastrointestinal parameters (bowel movement on auscultation, time to full oral nutrition, time to first defaecation, time to first flatus)
- Adverse events and adverse reactions
- Check of discharge criteria

* laboratory measurements in blood/serum samples 1st post-operative day:
  - in the morning sample: Troponin or high-sensitivity Troponin, NT-Pro-BNP, IL6 (facultative), IL10 (facultative), PCT, CRP, WBC, creatinine, BUN, PT, aPTT, vWF-Ag, vWF-CoRi, ROTEM
  - every 6 hours: blood gas analysis/acid base status/electrolytes (arterial or venous blood sample). Blood sampling for BGA should be performed as close as possible to the time, at which the corresponding hemodynamic measurement is done.
  - additional for injury scores: bilirubin, thrombocytes, haematocrit

***laboratory measurements in urine sample: creatinine, α1-MG,ß-NAG

day +2 – **+n**, until fulfillment of “fit for discharge”-criteria (Marshall, Chung 1997, 1999) however not longer than day +5 (including day +5) (*blood sampling also on postoperative day +3 and +7*)

- Haemodynamics (SAP, DAP, MAP, HR, CVP(every 6 hours); PiCCO: CI, SV, SVR; ITBVI, EVLWI (every 12 hours))
- Ventilatory support (PCV, CPAP) as requested to maintain normocapnia and oxygen saturation
- Blood sampling/urine sampling for laboratory evaluation (daily)*
- SIRS-criteria
- NuDesc (3x daily)
- Injury scores (APACHE II, SAPS II, SOFA) (once daily)

- Concomitant medication, infusion therapy (separate for colloid (gelatine) and crystalloid, blood products)
- Diuresis (sampling period from morning until next morning about 6 o'clock)
- PONV
- Surgical complications (e.g. anastomotic insufficiency, intestinal leakage, wound healing)
- Gastrointestinal parameters (bowel movement on auscultation, start of enteral nutrition, time to first defaecation)
- Mobilisation
- "Fit for discharge"-criteria (Marshall, Chung, 1997, 1999) (once on ICU-preferably in the morning)
- Aldrete Score (assessment at time when discharge from ICU)
- Adverse events and adverse reactions
- check of discharge criteria

*laboratory measurements on ICU

- once daily on ICU (maximally until day 5 (including day 5): renal function parameters (blood/serum: creatinine, BUN; urine: creatinine, α1-MG, ß-NAG), WBC
- additonal for injury scores: arterial pH, haematocrit, Sodium, Potassium, HCO3, bilirubin, platelets
- postoperative days 3 and 7: creatinine in serum

day +2 – day +15 maximal

- control at subsequent day whether the patient receives or not parenteral nutrition. Control of nutrition is stopped when the patient does not receive any parenteral nutrition and is able to take nutrients via the oral pathway (not enteral) at least 3 times that day.

3 months after surgery (telephone call only)

- Health Related Quality of Life (HRQoL)

14.2 Tabular Overview

TIME / PROCEDURE	**day -1** (range -5): inclusion visit / Pre-operative	**day 0**:prior anaesthesia/set perdural catheter	**day 0**: Pre-surgery (after induction of anaesthesia) set of arterial line, CVK	**day 0**: During surgery	**day 0**: At end of surgery	**day 0**: on admission to ICU	**day +1**: on ICU - 1st day post-op	**day +2 – days +n*:**	**At discharge** from ICU/MCU	**3 months after surgery (by phone)**
Written informed consent	x									
Inclusion / exclusion criteria	x									
History, demographics body weight + height	x						body weight			
Randomisation	x									
HRQoL	x									x
Body temperature			baseline x				x	x	x	
Haemodynamic parameters[1]			baseline x	every 15 min[9]	x	every 6 hours	every 6 - 12 hours	every 6 - 12 hours		
oesophageal probe/Doppler measurements CI, SV, SVR			baseline after final postiion	every 15 min	x					
Blood sample for:	see also separate table for laboratory parameters									
blood gases acid-base [2] electrolytes	Hct[8]		baseline x	every 60 min	x	every 6 hours	every 6 hours	x[16]		
haemostasis[3]	x[8]		baseline x	x[10]	x		x[13]	x[17]		
inflammatory[4]			baseline x		x[11]		x	x[18]		
renal function[5]	x[8]		baseline x					1x/d[19]	x[20]	
liver function	x[8]						x[14]	x[14]		
cardiac function[6]			baseline x		x		x[15]			
Urine sample for:	see also separate table for laboratory parameters									
pregnancy testing	x[8]									
renal function[7]			baseline x				x	1x/d	x	
Baseline administration of crystalloid		x	x	x						
Administration of IPs			after baseline measure ments x	x	x					
Intraabdom pressure			baseline x		x	6h after end of surgery 12	x[12, 15]			

PROCEDURE \ TIME	**day -1** (range -5): inclusion visit / Pre-operative	**day 0**:prior anaesthesia/set perdural catheter	**day 0**: Pre-surgery (after induction of anaesthesia) set of arterial line, CVK	**day 0**: During surgery	**day 0**: At end of surgery	**day 0**: on admission to ICU	**day +1**: on ICU - 1st day post-op	**day +2 – days +n*:**	**At discharge** from ICU/MCU	**3 months after surgery (by phone)**
Surgery data				x	x					
Concomitant medication			x	x	x	x	x	X Parent. Nutrition (max) til day 15	x	
Urinary output				x	x	x	x	x		
Blood products administration	x if requ.		x acc. trigger	x acc. trigg	x acc. trigg					
Blood losses				x	x					
Adverse events and adverse reactions		x	x	x	x	x	x	x	x	x
PONV						x	x	x		
Ventilatory support/ respiratory rate			x			x if requ	x if requ	x if requ		
ALDRETE Score						x	x[15]		x	
NuDesc/SIRS							x	x		
Injury Scores (Apache II incl Glasgow coma scale, SAPS II, SOFA)							x	1x/d		
Gastrointestinal parameters							x	x		
fit for discharge criteria fulfillment							x	x	x	

1 = SAP, DAP, MAP, HR; PiCCO: CVP, CI, SV, SVR

(see also oesophageal Doppler measurements of CI, SV, SVR for guidance of volume replacement therapy, i. e. infusion of Investigational Products)

2= Blood gases ((pCO2, pO2, HCO3, SaO2), acid-base status (pH, base excess, bicarbonate, lactate), electrolytes (sodium, potassium, ionized calcium, chloride)

3= PT, aPTT, Fibrinogen, vWF-AG, vWF-RiCo, FVIII:c, CT, CFT, MCF with ExTEM, FibTEM

4= PCT, CRP, WBC, IL6 (facultative), IL10 (facultative); baseline also HbA1

5= creatinine, BUN (BUN will be calculated from serum urea which will be measured)

6= Troponin or high-sensitivity Troponin, NT-Pro-BNP

7= α-1MG, ß-NAG, creatinine

8 = for evaluation of exclusion criteria (which includes haematocrit (venous blood), renal function/serum creatinine, hepatic function/MELD, Child-Pugh, electrolyte status/ Na, K, Ca)

9= every 60 minutes: EVLWI, ITBVI (PiCCO)

10= 1 hour after start of surgery: ROTEM: CT, CFT, MCF (ExTEM and FibTEM)

11=facultative IL6, facultative IL10

12= including pain assessment with VAS

13=1st postoperative day in the morning: PT, aPTT, ROTEM, vWF-AG, vWF-RiCo

14= bilirubin for score

15= 1st postoperative day in the morning

16= arterial pH, HCO3, sodium, potassium, Hct

17= platelets (for injury score)

18= WBC only for Scores

19= also on day 3 and 7 post-surgery: serum creatinine

20= serum creatinine

***Time n usually (if not otherwise mentioned) is defined as time when patient ready for discharge (exception of nutrition control) maximally until postoperative day 5 (including POD 5)**

Tabular overview of blood and Urine Sampling and Parameters

TIME	**day -1** (range -5) inclusion visit / Pre-operatively	**day 0** prior anaesthesia/set perdural catheter	**day 0** pre-surgery (after induction of anaesthesia)	**day 0** during surgery	**day 0** at end of surgery	**day 0** on admission to ICU	**day +1** on ICU - 1st day post-op	**day +2 - +n** on ICU	(postop.) **days +3** and **+7 and at discharge**	**3 months after surgery**
**Blood sample for:**										
*blood gas*es incl. [2] acid-base electrolytes Hkt	excl. criteria x		baseline x	every 60 min	x		every 6 hours	x[16]		
haemostasis										
PT	excl. criteria x		baseline x		x		in the morning x			
aPTT			baseline x		x		in the morning x			
Fibrinogen			baseline x		x					
vWF-AG			baseline x		x		in the morning x			
vWF-Ristocetin-Cofactor			baseline x		x		in the morning x			
FVIIIc			baseline x		x					
ROTEM (ExTEM, FibTEM): CT,CFT, MCF			baseline x	1 h after start x	x		in the morning x			
Platelets							x	x		
inflammatory										
facultative: IL6			baseline x		x		in the morning x			
facultative: IL10			baseline x		x		in the morning x			
PCT			baseline x				in the morning x			
CRP			baseline x				in the morning x			
WBC			baseline x				in the morning x	1x/d x		
renal function										
creatinine	excl. criteria x		baseline x				in the morning x	1x/d x	x	
HBA1c			baseline x							

TIME	**day -1** (range -5) inclusion visit / Pre-operatively	**day 0** prior anaesthesia/set perdural catheter	**day 0** pre-surgery (after induction of anaesthesia)	**day 0** during surgery	**day 0** at end of surgery	**day 0** on admission to ICU	**day +1** on ICU - 1st day post-op	**day +2 - +n** on ICU	(postop.) **days +3** and **+7 and at discharge**	**3 months after surgery**
BUN			baseline X				in the morning X	1x/d X		
liver function										
Albumin	excl. criteria X									
Bilirubin	excl. criteria X						X	X		
cardiac function										
Troponin or high-sensitivity Troponin			baseline X		X		in the morning X			
NT-Pro-BNP			baseline X		X		in the morning X			
**Urine Sample for:**										
α1-MG			x				x	x		
β-NAG			x				x	x		
Creatinine			x				x	x		

14.3 Assessment of Compliance

not applicable

14.4 Precautionary Measures

The following measures are listed in the repective SmPCs:

Severely dehydrated patients should receive intravenous electrolyte solutions first. Volume overload due to overdose should always be avoided. The dosage should be adjusted carefully. Severe haemodilution resulting from high doses of HES solutions should also be avoided in the treatment of hypovolaemic patients. (See section 11 for trigger of administration of blood products.) Elderly patients, who are more likely to suffer from cardiac insufficiency and renal impairment, should be closely monitored during treatment, and the dosage should be carefully adjusted, in order to avoid cardiocirculatory and renal complications resulting from hypervolaemia.

Further precautions as mentioned in the SmPc will not be mentioned in this section of the study protocol as respective patients are not to be included in the study: REFER TO SECTION 9.3 FOR CRITERIA OF PATIENTS WHO ARE *NOT ELIGILBLE* FOR PARTICIPATING IN THE PRESENT STUDY!

Because of the risk of allergic (anaphylactoid) reactions, the patient should be monitored closely.

Transiently raised alpha-amylase levels can occur after administration of solutions with HES. This should not be interpreted as a sign of pancreatic injury.

Because of the presence of calcium care should be taken to prevent extravasation during intravenous infusion. The solution should be given cautiously to patients with diseases associated with elevated vitamin D concentrations such as sarcoidosis. In case of concomitant blood transfusion, the solution must not be administered via the same infusion set. Solutions containing metabolisable anions should be administered cautiously to patients with respiratory impairment.

15 DURATION OF STUDY AND STUDY SCHEDULE

15.1 Duration of study per patient

Start of study: with randomisation

Treatment period (infusion of Investigational Products):

During surgery (guided by oesophageal Doppler measurements)

End of study: Postoperative day 15 or hospital discharge, whatever occurs first

Last evaluation of certain parameters will be 3 months post-surgery respectively when DRG data are available.

15.2 Study Schedule: Duration of whole study

Planned start:	June 2010
Planned recruitment time:	2 years
Planned last patient out:	May 2012

16 STATISTICS

16.1 Statistical methods

This section gives an overview of the statistics planned for the study. All programming of tables, figures, listings and statistical analyses will be performed using the statistical software packages SPSS® version 13, SAS® version 9.1.3 and StatXact® version 6.

A Statistical Analysis Plan (StAP) will be finalized before close of database. It includes all protocol amendments. In case a protocol amendment is passed after finalization of the StAP an amendment to the StAP is to be composed.

All target criteria (primary and secondary variables) will first be examined by exploratory data analysis and descriptively evaluated (Wernecke 1995). In this setting, the evaluation of structural homogeneity of the treatment groups will be performed for the purpose of quality assurance.

A multiple primary endpoint consisting of “amount of HES-solution in ml” (first primary variable) and “days until fully on oral (solid) diet” (second primary variable) is defined. The principle of ordered hypothesis will be used (Hothorn and Lehmacher, 1991)(Maurer et al., 1995) for testing the two primary efficacy parameters. If the first primary hypothesis will result in a statistically significant difference of the amount of the hyperoncotic HES-solution as compared with the amount of iso-oncotic HES-solution, the second primary variable will be analyzed using the same procedure (with an unchanged error of the 1st kind $\alpha = 5\%$) as described for the frist primary variable.

The two primary variables will be evaluated with a non-parametric statistical test (Mann-Whitney U-test, Hartung and Elpelt, 1993) taking into consideration small sample sizes and possible deviation from normal distribution. Stratification variables will be included in the primary analysis as covariates (in a nonparametric analysis of covariance cf. Bathke, Brunner 2003).

Secondary target variables will also be evaluated with nonparametric tests according to their scaling. Whereby, in case of a small random sample size or an unbalanced condition, exact tests will be used (Mehta, Patel, 1998).

Because the study extends over a longer time period and during this time multiple readings/measurements will be taken from the patients, it is necessary to analyze the progress of the primary target criterion and further clinical parameters over the entire study period. Therefore, a non-parametric multivariate analysis of variance (MANOVA) (Brunner *et al*. 2002) for repeated measurement will be performed.

16.2 Level of significance and power

All tests will be conducted with an error of 1st kind α = 5%, two-sided.

Tests of secondary variables will be carried out in the area of exploratory data analysis. Therefore, corresponding p-values are to be regarded as exploratory ones and no adjustments for multiple testing will be made.

Sample size calculations are to be performed with a power of 80%

16.3 Statistical hypotheses

The primary objective is the efficacy of target controlled fluid therapy with a hyper-oncotic compared to an iso-oncotic balanced HES 130/0.42 solution, so that the following hypotheses need to be tested

for the first primary variable (amount of HES-solution [VR]):

H_{01} (null hypothesis):
VR [hyperoncotic HES-solution] = VR [iso-oncotic HES-solution]

H_{A1} (alternative hypthesis, two-sided):
VR [hyperoncotic HES-solution] $\neq$ VR [iso-oncotic HES-solution]

and for the second primary variable (days until fully on oral diet [DD]):

H_{02} (null hypothesis):
DD [hyperoncotic HES-solution] = DD [iso-oncotic HES-solution]

H_{A2} (alternative hypthesis, two-sided):
DD [hyperoncotic HES-solution] $\neq$ DD [iso-oncotic HES-solution]

16.4 Sample Size

The first primary variable, i. e. amount of plasma volume replacement therapy (in addition to a basal electrolyte infusion), will serve to calculate sample size. It is assumed that 250ml difference between the two HES treatment arms is clinically relevant.

It is roughly calculated that 228 patients shall be included which is based on the following assumptions: 69 patients per group, supposing an effect size (difference of means/(common standard deviation) = ½ = 0.5, error of the 1st kind alpha = 5% (two-sided), Power = 80% and the nonparametric Mann-Whitney U-Test (Calculations with

nQuery Advisor, version 6.0). Taking the drop-out rate of 10% into consideration, a sample size of 76 patients per group will be needed.

However, at present no reliable information is available on the amount of plasma volume requirement in elective pancreatic surgery. Thus, an internal pilot phase including 60 patients (including drop-outs) will be performed in order to re-calculate total sample size. For re-calculation the pooled variance will be assessed on the grounds of clinical relevant difference without unblinding. Type I error for both phases of the study will strictly be maintained. (Kieser and Friede, 2000 and 2003)

For the second primary endpoint it is assumed that 1.5 day difference between the two treatment arms will be achieved. Based on hospital's own data an effect size (difference of means/ common standard deviation) of 0.698 is calculated which reveals that inclusion of 69 patients – as for the first primary endpoint (without dropouts) – will lead to a power of 97%. A sample size of only 37 (per group) is demanded to achieve a power of 80%. Therefore, enough power is ensured to prove the second primary endpoint after significance of the first. After pilot phase, the re-estimation of the (pooled) variance will also be accomplished for the second primary variable in order to ensure a sufficient power comparing the two treatments with the former re-calculated sample size (see above).

16.5 Data handling

All data will be listed. Whenever applicable all tables, figures and listings will identify patients using the patient identification (i. e. randomisation-number) and time of evaluation. For data collected prior to randomization, this will be done likewise (i. e. use of subsequently obtained randomisation number)

The actual population presented in a table/figure/listing will be mentioned in the headings.

Tables and listings will be produced in accordance with the principles outlined by the ICH E3 guideline.

Handling missing data and outliers

Every effort will be made to collect all data points in the study. The amount of missing data will be minimized by appropriate management of the randomized, prospective, trial, proper screening of subjects, and training of participating investigators and other authorized staff (e.g. nurses), monitors and study co-ordinator. Since all patients who are randomized will be included in the primary analysis, in those instances where data are missing, missing values will not be imputed.

Missing data in tables and listings will be handled as missing data by inserting the symbol '.'.

Outliers may be identified using stem-leaf plots and frequency distributions, Scatter and box plots may be also generated for outlier identification. For normally distributed data, values more than three standard deviations away from the mean will be considered outliers. Transformation of the data to mitigate the influence of outliers may be considered. If outliers remain, additional analyses excluding these values will be done and discussed in the report.

16.6 Interim Analysis

Not applicable.

The confirmation respective recalculation of sample size for the first primary variable and assurance of power for the second primary variable after inclusion of 60 patients will only be performed by estimating the pooled variance (without unblinding). (Refer to section 16.4)

16.7 Criteria for the termination of the study

If after pilotphase (after 60 patients) the calculation of the pooled variance reveals that the estimated total number of 228 patients will be exceeded the study will not be carried on but stopped for futility after the pilot phase.

16.8 Patient selection for analyses

An Intent-To-Treat (ITT) analysis and Full Analysis Set (FAS) respectively is planned as primary.

Additionally, an Per-Protocol (PP) or Valid Case Analysis Set (VCAS) respectively will be performed i. e. all patients where none of the following events occurred:

- Stop of treatment due to adverse reaction
- severe violation of study protocol

The results of both analyses will be compared and possible differences have to be discussed accordingly.

The definitions for any population groups chosen for analysis (e.g. all patients randomized, intent-to-treat, per-protocol, completers) will be provided in the statistical analysis plan. Decision on analysis of the respective study population will be performed prior unblinding.

17 SOURCE DATA AND SOURCE DOCUMENTS

17.1 Definitions

17.1.1 Source Documents

Source documents are defined as original documents, data and records (e.g. hospital records, clinical and office charts, laboratory notes, memoranda, patient diaries or evaluation check lists, pharmacy dispensing records, recorded data from automated instruments, copies or manuscripts certified after verification as being accurate copies, microfiches, photographic negatives, microfilm or magnetic media, x-rays, patient files, records kept at pharmacy, at the laboratories and at medico technical departments involved in clinical study).

Refer to section 12.5 which documents serve as source documents for the present study.

17.1.2 Source Data

Source data are defined as all information in original records and certified copies of original records of clinical findings, observations or other activities in a clinical study necessary for the reconstruction and evaluation of the study. Source data are contained in source documents (original records or certified copies).

17.1.3 Direct Access

Direct access is defined as the permission to examine, analyse, verify and reproduce any records and reports that are important to evaluation of a clinical study.

17.2 Permission of Access

The Investigator will permit study-related monitoring, audits, IRB / IEC review and regulatory inspections, providing direct access to primary patient data (i.e. source data) which supports the data on the CRFs for the study, e. g. general practice charts, hospital notes, appointment books, original laboratory records etc.

Because this enters into the realm of patient confidentiality, this fact must be included in the Informed Consent Form to be signed by the patient, in line with pertinent data protection legislation.

Any party (e.g. domestic and foreign regulatory authorities, the Sponsor and / or authorised representatives of the Sponsor such as monitors and auditors) with direct access should take all reasonable precautions within the constraints of the applicable regulatory requirements to maintain the confidentiality of patient identities and Sponsor proprietary information.

18 QUALITY CONTROL AND QUALITY ASSURANCE

18.1 Quality Control

18.1.1 Definition

Quality Control is defined as the operational techniques and activities, such as monitoring, undertaken within the quality assurance system to verify that the requirements for quality of the study related activities have been fulfilled.

Quality Control should be applied to each stage of data handling to ensure that all data are reliable and have been processed correctly.

18.1.2 Study Monitoring

Authorized, qualified representatives of the Sponsor will visit investigational sites in regular intervals as defined in the monitoring plan to verify adherence to protocol and local legal requirements, to perform source data verification and to assist the Investigator in his study related activities.

Refer also to section 17.2.

18.2 Quality Assurance

18.2.1 Definition

Quality Assurance is defined as the planned and systematic actions that are established to ensure that the study is performed and the data are generated, documented (recorded) and reported in compliance with Good Clinical Practice (GCP) and the applicable regulatory requirements.

18.2.2 Audit

An audit is a systematic and independent review of study related activities and documents to determine whether the evaluated study related activities were conducted and the data were recorded, analysed and accurately reported according to the protocol, designated Standard Operating Procedure (SOPs), Good Clinical Practice (GCP) and the applicable regulatory requirements. An independent audit at the study site may take place at any time during or after the study.

Refer also to section 17.2.

18.2.3 Inspection

An Inspection is defined as the act by a regulatory authority of conducting an official review of documents, facilities, records and any other resources that are deemed by the authorities to be related to the clinical study and that may be located at the site of the study, or at the Sponsors and / or clinical research organisation facilities or at any other establishments deemed appropriate by the regulatory authorities.

Refer also to section 17.2.

19 ETHICAL AND LEGAL CONSIDERATIONS

19.1 Committees and Boards

19.1.1 Independent Ethics Committee (IEC)

This is an independent body (a review board or a committee, institutional, regional, national or international) constituted of medical / scientific professional and non-medical / non scientific members whose responsibility it is to ensure the protection of the rights, safety and well being of human subjects involved in the study, and to provide public assurance of that protection, by reviewing and providing a favourable opinion on the study protocol, suitability of the Investigator, facilities and the methods and material to be used in obtaining and documenting informed consent from study patients

The legal status, composition, function, operations and regulatory requirements pertaining to the Independent Ethics Committee may differ among countries, but should allow the Ethics Committee to act in agreement with GCP.

Regarding the conduct of the present study and involvement of IEC refer to section 19. 2.

19.1.2 Institutional Review Board (IRB)

This is an independent body constituted of medical scientific and non-scientific members, whose responsibilities is to ensure the protection of the rights, safety and well being of human subjects involved in a study by reviewing approving and providing continued review of the study protocol and amendments and of the methods and material used in obtaining and documenting informed consent of the study patients.
An IRB is not applicable for the present study.

19.1.3 Drug Safety Monitoring Board (DSMB)

A Data Monitoring Board/Committee is a group of independent experts external to a study assessing the progress, safety data and, if needed critical efficacy endpoints of a clinical study. In order to do so a DSMB may review unblinded study information (on a patient level or treatment group level) during the conduct of the study. Based on its review the DSMB provides the sponsor with recommendations regarding study modification, continuation or termination.

For assessment of the need of a DSMB in a respective study refer to EMEA/CHMP/EWP/5872/03 Corr.

For the present phase IV study a DSMB will not be appointed.

19.2 Conduct of Study and Ethical Considerations

This clinical study will be conducted in accordance with the Declaration of Helsinki (see Appendix 1). It will be conducted in compliance with this protocol, Good Clinical Practice (2001/20/EEC, CPMP/ICH/135/95), designated SOPs, and with local laws and regulations relevant to the use of investigational new drugs in the country of conduct.

Before initiating a study, the Investigator should have written and dated approval / favourable opinion from the concerned IEC for the study protocol (and any amendments), written informed consent form, consent form updates, patient recruitment procedures (e.g. advertisements), and any other written information to be provided to patients. Approval will be indicated in writing with reference to the final protocol number and date. Details of the IEC's constitution including names of its members and their function in the committee (e.g. chairman, specialist, lay-member) should be made available to the Sponsor for inclusion in the Trial Master File.

During the study all documents that are subject to review should be provided to the IEC by the sponsor or the Investigator in line with national provisions.

19.3 Responsibilities

The responsibilities of the Investigator, Monitor and Sponsor of the clinical trial as regards handling of data, storage of data, planning, assessment and quality assurance are regulated by the recommendations on " ICH Topic E 6 Guideline for Good Clinical Practice" of the "International Conference on Harmonisation" (ICH) and apply also to this clinical trial.

19.4 General reporting obligation

B. Braun AG shall apply for the authorization of the clinical study at the federal authority and inform local authorities responsible for the Investigators and for the company itself about the planned clinical trial in writing before the beginning of the study.

19.5 Financing and Insurance

The costs necessary to perform the study will be agreed upon with each Investigator and will be documented in a separate financial agreement which will be signed by the Investigator and the Sponsor, prior to the study commencing.

The B. Braun AG has taken out subject insurance with a company of the for all patients taking part in the trial under the

policy number

All investigators shall receive a copy of the insurance certificate and the insurance conditions; the latter must be known to the patients and made available on request.

19.6 Personal Data and Data Protection

All data obtained in the context of the clinical trial are subject to data protection. The patient's name in addition to other personal data (except demographic data like age and sex) are not to be disclosed by the Investigator.

It must be ensured that CRFs or other documents (e.g. copies of reports on special findings) transmitted to the B. Braun AG contain no names, but only the patients' study identification (i. e. random number).

The storage of data for statistical assessment shall likewise be performed only under the patient's study identification. Only the Investigator will have the means to identify a patient's name / other personal details via the study identification.

If it becomes necessary in the course of the study to identify a patient's name for medical reasons, all individuals involved are subject to an obligation to maintain secrecy.

If personal data are stored and processed, the requirements of pertinent data protection legislation are to be observed.

19.7 Modification of Protocol

The Investigator or the Sponsor should not implement any deviation from, or changes of, the protocol without mutual agreement, prior review and documented approval from the IEC of a respective amendment. The only exceptions are where necessary to eliminate an immediate hazard to study patients, or when the changes involve only logistical or administrative aspects of the study (e.g. change in monitor(s), change of telephone number(s)).

The party initiating an amendment must confirm it clearly in writing and it must be signed and dated by the Sponsor and the Principal Investigator. Protocol amendments will be submitted to the concerned IECs and competent authorities in line with pertinent requlatory requirements.

19.8 Investigator's Brochure/Summary of Product Characteristics

The Investigator shall be informed about the preclinical and clinical state of knowledge concerning the Investigational Products.

In the present study this will be done by means of the approved SmPC. This document should serve as the basis for the assessment of expectedness of an adverse reaction (see section 11.1.3).

19.9 Completion of Case Report Forms

Any data to be recorded directly on the CRFs (to be considered as source data) will be defined at the start of the study (see section 12.5).

For paper CRFs all entries should made in black ink for duplication purposes. The investigator must ensure the accuracy, completeness, legibility and timeliness of data reported in the CRF and all required reports.

In case electronic CRFs (remote data entry) are used the investigator must strictly adhere to the written instructions of the respective software description.

Any change or correction to a CRF must be dated, initialled and explained (if necessary) and must not obscure the original entry, this applies to both written and electronic changes.

For the present study considerations regarding the use of electronic CRFs are made. In case this applies, the software of the CRO as mentioned in section 1 in the current available version will be used. Details on the software and version number will be compiled in a separate document prior to start of the study and filed in the study files.

Data reported on the CRF that are derived from source documents should be consistent with the source documents or the discrepancies should be explained.

For paper CRFs the Investigator should agree to have completed and signed CRFs available for full inspection by the clinical monitor within two weeks after completion of each patient.

19.10 Archiving

Essential documents are to be retained for the periods required by ICH-GCP, i. e. until at least 2 years after the last approval of a marketing application in an ICH region and until there are no pending or contemplated marketing applications in an ICH region or at least 2 years have elapsed since the formal discontinuation of clinical development of the Investigational Product (CPMP/ICH/135/95), or by national legal requirements, whichever is longer, but not less than 15 years after routine/premature termination of a clinical study.

The final report shall be retained for at least 2 years after the Investigational Products are removed from the last market.The informed consent forms and all the original (raw) data are to be retained by the investigator for at least 15 years.

19.11 Confidentiality

The aim and contents of the study, in addition to its results are to be treated as confidential by all persons involved in the clinical trial.

20 FINAL REPORT AND PUBLICATION POLICY

The Sponsor and Investigator shall agree on the final study report.

It is intended that the results of the study may be published as scientific literature. Results may also be used in submissions to regulatory authorities. The following conditions are to protect commercial confidential materials (patents, etc), not to restrict publication.

All information concerning Tetraspan® or Sterofundin® ISO (such as patent applications, formulae, manufacturing processes, basic scientific data, or formulation information supplied to the Investigator by the Sponsor and not previously published) is considered confidential and shall remain the sole property of the Sponsor. The Investigator agrees not to use it for other purposes without the Sponsor's written consent.

It is understood by the Investigator that the Sponsor will use the information developed in this clinical study in connection with the development of Tetraspan® and therefore may be disclosed as required to other Investigators or any appropriate international Regulatory Authorities. In order to allow for the use of information

derived from this clinical study, the Investigator understands that he/she has an obligation to provide the Sponsor with complete test results and all data developed during this study.

Prior to submitting the results of this study for publication or presentation, the Investigator will allow the Sponsor 30 days in which to review and comment upon the publication manuscript. The Sponsor agrees that before he publishes any results of this study, he shall provide the Investigators at least 30 days for full review of the publication manuscript. In accordance with generally recognised principles of scientific collaboration, co-authorship with any Sponsor personnel will be discussed and mutually agreed upon before submission of a manuscript to a publisher.

21 References

Agency for Healthcare Research and Quality. (2007) Esophageal Doppler Ultrasound-Based Cardiac Output Monitoring for Real-Time Therapeutic Management of Hospitalized Patients. A Review.

Aldrete JA. (1995): The post anaesthesia recovery score revisited [letter]. J Clin Anesth;7:89 – 91

Bathke, A. and Brunner, E. (2003). A Nonparametric Alternative to Analysis of Covariance. In: M.G. Akritas and D.N. Politis (eds.): Recent Advances and Trends in Nonparametric Statistics. Amsterdam. Elsevier B.V. pp 109-120.

Boldt,J. (2005) Volume Therapy in Cardiac Surgery. Annals of Cardiay Anaesthesia, 8, 104-116.

Boldt,J. & Suttner,S. (2005) Plasma Substitutes. Minerva anestesiologica, 71, 741-758.

Bone RC et al (1992): Definitions for sepsis and organ failure and guidelines for the use of innovative therapies in sepsis. The ACCP/SCCM Consensus Conference Committee. Chest 101 (6): 1644–55

Brandstrup,B. (2006) Fluid Therapy for the Surgical Patient. Best practice & research.Clinical anaesthesiology, 20, 265-283.

Brunner, E., Domhof, S. and F. Langer (2002): Nonparametric Analysis of Longitudinal Data in Factorial Experiments. *Wiley & Sons, New York*

Hartung, K. und Elpelt, B. (1993): Statistik. Oldenbourg, 9. Auflage, München

Hothorn, L. & Lehmacher, W (991) A Simple Testing Procedure „Control versus k Treatments“ for One-sided Ordered Alternatives, with Application in Toxicology. Biometrical Journal, 33 (2) 179-189

Jungheinrich,C. & Neff,T.A. (2005) Pharmacokinetics of Hydroxyethyl Starch. Clinical pharmacokinetics, 44, 681-699.

Kieser, M. and Friede, T. (2000): Blinded sample size reestimation in multiarmed clinical trials. *Drug Information Journal*. **34**, 455-460

Kieser, M., Friede, T. (2003): Simple procedures for blind sample size adjustment that do not affect the type I error rate. *Statistics in Medicine* **22**; 3571-3581

Klein M. (2005): Dissertation Jena

Knaus WA et al. (1985): APACHE II: a severity of disease classification system. Crit Care Med.; 13; 818-29.

Le Gall JR et al. (1993): A new simplified acute physiology score (SAPS II) based on a European / North American multicenter study. JAMA;270; 2957-63

Lütz A et al. (2008): Nursing Delirium screening Scale - Richtlinienkonforme Übersetzung für den deutschsprachigen Raum. Anästhesiol. Intensivmed. Notfallmed. Schmerztherapie; 2; 98 – 102

Lobo,D.N., Macafee,D.A., & Allison,S.P. (2006) How Perioperative Fluid Balance Influences Postoperative Outcomes. Best practice & research.Clinical anaesthesiology, 20, 439-455.

Marshall S. I., F. Chung (1999): Discharge criteria and complications after ambulatory surgery. Anesth.Analg.; 88; 508 – 17

Marshall S, Chung F. Assessment of "home readiness": discharge criteria and postdischarge complications. Curr Opin Anaesthesiol 1997;10:445–50.

Maurer, W., Hothorn, L. & Lehmacher, W (1995) Multiple comparisons in drug clinical trials and preclinical assays: a-priori ordered hypotheses. In: Biometrie in der chemisch-pharmazeutischen Industrie. Fischer, Stuttgart, Volume 6, 3-18

Mehta, C. R. and Patel, N. R. (1998): Exact inference for categorical data. In: P. Armitage, and T. Colton (eds.): Encyclopedia of Biostatistics. New York, Wiley & Sons. pp 1411-1422.

Noblett SE, Snowden CP, Shenton BK, Horgan AF.(2006) Randomized clinical trial assessing the effect of Doppler-optimized fluid management on outcome after elective colorectal resection. *Br J Surg*. **93(9)**:1069-76.

Vincent et al. (1996): The SOFA (Sepsis-related Organ Failure Assessment) score to describe organ dysfunction / failure. Intens. Care Med.; 22; 707-710

Wakeling HG, McFall MR, Jenkins CS, Woods WG, Miles WF, Barclay GR, Fleming SC. (2005) Intraoperative oesophageal Doppler guided fluid management shortens postoperative hospital stay after major bowel surgery. *Br J Anaesth.* **95**(5):634-42

Wernecke, K.-D. (1995): Angewandte Statistik für die Praxis. Addison-Wesley Publishing Comp., Bonn, Paris

22 SIGNATURES

The undersigned have read this protocol and agreed to conduct this study in accordance with all stipulations of the protocol and in accordance with the Declaration of Helsinki.

Date	Signature
______________________	.... (Study Coordinator B. Braun)
______________________	.... (Head Clinical Development B. Braun)
______________________	.... (Chief Medical Officer B. Braun)
______________________	.... (Statistician)
______________________	.... Coordinating Investigator
______________________	.... Principal Investigator
______________________	.... Principal Investigator
______________________	.... Principal Investigator

23 APPENDICES

1 Declaration of Helsinki (*version dated 1996*)

2 Patient Information Sheet and Informed Consent Form

3 Screening Log

4 Enrolment Log

5 Randomisation Sheet

6 Dosing Algorithm

7 Inventory Form

8 NuDesc and Scores, Fit for Discharge-criteria

9 Serious Adverse Event Form
